# Dopaminergic dynamics underlying sex-specific cocaine reward

**DOI:** 10.1038/ncomms13877

**Published:** 2017-01-10

**Authors:** Erin S. Calipari, Barbara Juarez, Carole Morel, Deena M. Walker, Michael E. Cahill, Efrain Ribeiro, Ciorana Roman-Ortiz, Charu Ramakrishnan, Karl Deisseroth, Ming-Hu Han, Eric J Nestler

**Affiliations:** 1 Fishberg Department of Neuroscience, Friedman Brain Institute, Icahn School of Medicine at Mount Sinai, 1 Gustave L Levy Place, New York, New York 10029, USA; 2 Department of Pharmacological Sciences, Friedman Brain Institute, Icahn School of Medicine at Mount Sinai, New York, New York 10029, USA; 3 Department of Bioengineering, Stanford University, Stanford, California 94305, USA; 4 HHMI, Stanford University, Stanford, California 94305, USA; 5 Department of Psychiatry and Behavioral Sciences, Stanford University, Stanford, California 94305, USA

## Abstract

Although both males and females become addicted to cocaine, females transition to addiction faster and experience greater difficulties remaining abstinent. We demonstrate an oestrous cycle-dependent mechanism controlling increased cocaine reward in females. During oestrus, ventral tegmental area (VTA) dopamine neuron activity is enhanced and drives post translational modifications at the dopamine transporter (DAT) to increase the ability of cocaine to inhibit its function, an effect mediated by estradiol. Female mice conditioned to associate cocaine with contextual cues during oestrus have enhanced mesolimbic responses to these cues in the absence of drug. Using chemogenetic approaches, we increase VTA activity to mechanistically link oestrous cycle-dependent enhancement of VTA firing to enhanced cocaine affinity at DAT and subsequent reward processing. These data have implications for sexual dimorphism in addiction vulnerability and define a mechanism by which cellular activity results in protein alterations that contribute to dysfunctional learning and reward processing.

## Abstract (web summary)

Sex differences in reward processing are at present poorly understood. Calipari and Juarez *et al*. report oestrous cycle-dependent fluctuations in firing of VTA dopamine neurons that drive alterations in DAT function expressed in terminals in the NAc. These differences underlie enhanced cocaine reward processing during oestrus.

Drug addiction is a debilitating neuropsychiatric disorder characterized by high levels of drug intake, drug seeking and repeated cycles of abstinence and relapse^1^. Although both males and females become addicted to cocaine, females are reported to transition to addiction faster, take more cocaine, experience more adverse consequences and have more difficulty remaining abstinent in humans^2^. In the human literature, although males are more likely to be given the opportunity to try cocaine than females, both sexes progressed to regular use at an equal rate once exposed to cocaine^3^,^4^. Further, when opportunity for drug use is taken into consideration, female use exceeds that of males^4^,^5^. Women are also more likely to use cocaine at an earlier age^6^, take drugs in larger quantities^5^,^7^, report greater difficulty remaining abstinent^8^ and relapse^9^,^10^. Although these sex differences in humans could be caused by psychosocial and cultural factors, as well as neurobiological mechanisms, similar sex differences are observed in animal models and the precise neurobiological mechanisms of these sex differences in drug-reward processing remain poorly understood.

In addition to females displaying evidence for higher risk for addiction than males, there are also several reports of menstrual cycle-dependent fluctuations in the subjective effects of cocaine and drug craving in females^11^,^12^. In humans, both the cardiovascular and subjective responses to cocaine were reduced during the luteal phase of the menstrual cycle^13^, when oestrogen levels are declining. Conversely, during the follicular phase, when oestrogen levels are rising, women reported a greater ‘high' from cocaine administration^11^,^13^, suggesting a potential link between ovarian hormones and drug craving. Although the menstrual cycle has not definitively been linked to this process in humans, these reports nonetheless raise the possibility of sexually dimorphic mechanisms of reward processing and suggest that menstrual/oestrous cycle effects may be involved.

Circulating ovarian hormones, including oestrogens, act throughout the entire nervous system via non-genomic and genomic mechanisms. During development, X-chromosome genes play a role in developmental programming of the dopamine system via genetic and hormonal factors that can lead to sex differences in both basal function and the hormonal control over these processes^14^,^15^,^16^. Accordingly, multiple studies have shown sex differences in the ability of estradiol to stimulate dopamine release^17^,^18^, which could act to drive the increased reward learning that occurs during oestrus. Although these changes in reward learning that occur in oestrus can be viewed as adaptive to promote survival, the process might also lead to changes in drug reward, thus increasing drug abuse vulnerability. However, the precise mechanisms by which this occurs remain to be elucidated.

Studies in animal models have implicated ovarian hormone-dependent changes in drug reward functioning^19^. However, most of these investigations used ovariectomized females with hormone replacement to observe these effects, ablating the natural fluctuations in hormones observed with the oestrous cycle. To determine oestrous cycle-dependent, sexually dimorphic cellular and molecular mechanisms controlling cocaine reward, we used electrophysiology, fast-scan cyclic voltammetry (FSCV) and *in vivo* calcium imaging via fibre photometry to study the ability of male and naturally cycling, intact female mice in either dioestrus (low circulating hormones) or oestrus (high circulating hormones) to associate the rewarding effects of cocaine with the contextual cues that predict cocaine administration. By monitoring cycle stages of female mice, we determined how oestrous cycle-dependent fluctuations in reward system activity underlie reward processing and how this can influence addiction vulnerability. Here we outline a mechanism by which enhanced function of the dopamine system converges with increased pharmacological effects of cocaine to enhance these associations.

## Results

### Oestrus females form stronger cue-reward associations

**Figure 1:**
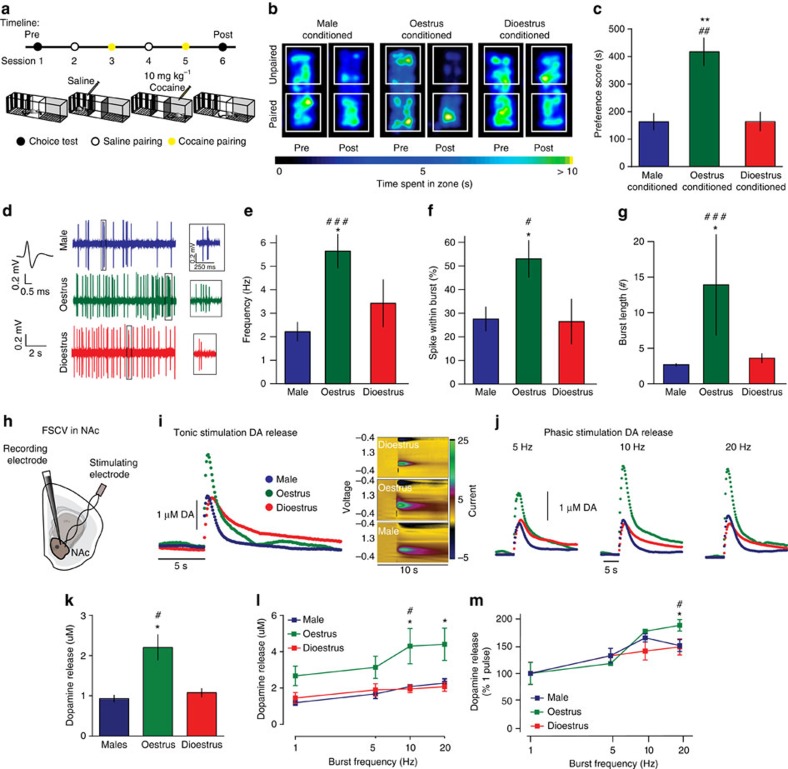
Oestrus-conditioned female mice exhibit elevated CPP for cocaine and enhanced basal reward circuit function. (**a**) Timeline of cocaine CPP experiments. (**b**) Representative heat maps of time spent in each area of the CPP chamber for male (left), oestrus-conditioned females (centre) and dioestrus-conditioned females (right). (**c**) Increased CPP in females was only observed in mice paired with cocaine conditioning during oestrus (one-way analysis of variance (ANOVA); F_(2, 12)_=13.13, *P*<0.001; **P<0.01 oestrus versus dioestrus, ^##^*P*<0.01 oestrus versus male). (**d**) Electrophysiological traces showing increased phasic activity of VTA dopamine neurons in oestrus females. *In vivo* single-unit electrophysiology of basal activity of putative VTA dopamine neurons identified increased: (**e**) firing rate (Kruskal–Wallis (KW) (*χ*^2^_(2)_=11.76, *P*<0.005; *P<0.05, ^###^*P<*0.001), (**f**) percentage of spikes within burst (one-way ANOVA F_(2, 36)_=7.858, *P*<0.005; */^#^*P<*0.05) and (**g**) burst length (KW (*χ*^2^_(2)_=10.17, *P*<0.01; *P<0.05, ^###^*P<*0.001) in only oestrus females when compared with males (#) or dioestrus females (*). (**h**) FSCV recordings of subsecond dopamine release in the NAc. (**i**) Current versus time plots (left) and colour plots showing the presence of dopamine after one pulse tonic stimulation, as indicated by its oxidation at +0.6 V and reduction at −0.2 V. (**j**) Current versus time plots showing increased dopamine release to increasing frequency of five pulse stimulations. (**k**) Group data showing enhanced NAc dopamine per one pulse tonic stimulation in oestrus females (one-way ANOVA; F_(2, 6)_=11.57; *P<0.05, ^#^*P<*0.05). (**l**) Phasic responsivity was increased in oestrus females (two-way ANOVA; F_(6, 18)_=3.279, *P*<0.05; *P<0.05, ^#^*P<*0.05). (**m**) Phasic responses represented as a percent of one pulse (tonic) release. Oestrus females exhibit increased dopamine release during phasic stimulations (two-way ANOVA; F_(3, 14)_=9.079, *P*<0.05; *P<0.05, ^#^*P<*0.05). *P<0.05, **P<0.01, ***P<0.001 oestrus versus dioestrus (unless otherwise noted); ^#^*P*<0.05, ^##^*P*<0.01 oestrus versus male. Data represented as mean±s.e.m.

We first conducted conditioned place preference (CPP) for cocaine, which provides an indirect measure of drug reward, in male and intact female C57BL/6J mice. Animals were injected with 10 mg kg^−1^ intraperitoneally (i.p.) cocaine and confined to one side of a three-chamber CPP apparatus. In alternating sessions, the other chamber was paired with saline (Fig. 1a). Vaginal cytology was performed daily to determine cycle stage for each female throughout the sessions. In a choice test 24 h after the final pairing, male and female mice were allowed to freely explore both chambers in the absence of drug.

Both male and female mice spent more time on the side that was previously paired with cocaine; however, female mice exhibited higher levels of place preference compared with males, indicating enhanced reward to this moderate cocaine dose (Supplementary Fig. 1). Interestingly, this enhanced cocaine reward was unique to female mice that were conditioned specifically during proestrus/oestrus, with female mice conditioned during dioestrus (dioestrus I and II) forming place preferences similar to those seen in male mice (Fig. 1b,c). These oestrous cycle-dependent conditioning effects indicate a unique mechanism by which cue-reward associations are enhanced selectively during oestrus.

### Oestrus females show enhanced dopamine function

Both drug reward and cue-reward associations (that is, associative learning) are dependent on dopaminergic transmission from the ventral tegmental area (VTA) to the nucleus accumbens (NAc)^20^. Phasic firing of VTA dopamine neurons is a critical component in associative learning between environmental cues and rewarding stimuli^21^. We thus wanted to determine whether there was differential basal VTA dopamine neuron activity and firing properties between male, oestrus female and dioestrus female mice.

Using anaesthetized, single-unit *in vivo* electrophysiology in drug-naive male mice, dioestrus female mice and oestrus female mice, we found that female mice overall exhibit increased basal putative VTA dopamine neuron activity when compared with male mice (Supplementary Fig. 2a–d). However, the increased putative VTA dopamine neuron firing rate was specific for female mice in oestrus (Fig. 1d,e). Furthermore, the percentage of spikes found in bursts and the burst lengths were also significantly higher in oestrus females than males and dioestrus females (Fig. 1d,f,g and Supplementary Fig. 2e). This is consistent with a previous report that female rats in oestrus displayed increased basal putative VTA dopamine neuron activity and bursting when compared with female rats in dioestrus^22^. Together, these data demonstrate a selective enhancement in basal neural activity of the reward system during oestrus.

Although phasic activity of VTA dopamine neurons is a critical mediator of reward encoding, ultimately dopamine release from nerve terminals in the NAc is the outcome that encodes this reward processing^23^. Studies using microdialysis have previously shown elevated dopamine tone in the NAc of oestrus females^24^; however, microdialysis is unable to distinguish between tonic and phasic dopamine levels to identify the temporal profile of dopaminergic responses. Using FSCV in the NAc in brain slices, we measured subsecond dopamine release that occurs in response to tonic and phasic stimulation of dopamine terminals originating in the VTA (Fig. 1h). We found that oestrus females, compared with both males and dioestrus females, release two times more dopamine to single-pulse tonic stimulations (Fig. 1i,k). Further, they are more responsive to phasic stimulation parameters (Fig. 1j,l), even after controlling for baseline differences in release (Fig. 1m).

Together, these data suggest that the dopaminergic VTA-NAc circuit of oestrus females is more active, even before cocaine conditioning. Increases in dopamine release can be due to increases in dopamine synthesis or in alterations in feedback mechanisms at the level of the nerve terminal. Levels of tyrosine hydroxylase (TH), the rate-limiting enzyme for dopamine synthesis, were not changed, nor was the phosphorylation of TH at any of the sites that alter its catalytic activity (Supplementary Fig. 3). However, D2-autoreceptor feedback mechanisms are greatly blunted in oestrus females as indicated by decreased sensitivity to a D2-receptor agonist, quinpirole (Supplementary Fig. 4a–c). This could underlie both the enhanced tonic release and the enhanced responsivity to phasic stimulations. Collectively, these data show that, specifically during oestrus, the reward system is primed to respond maximally to salient stimuli, an effect that could increase the likelihood of associations between rewards and predictive cues during this period.

### Fluctuations in the VTA-NAc circuit are oestrous dependent

**Figure 2:**
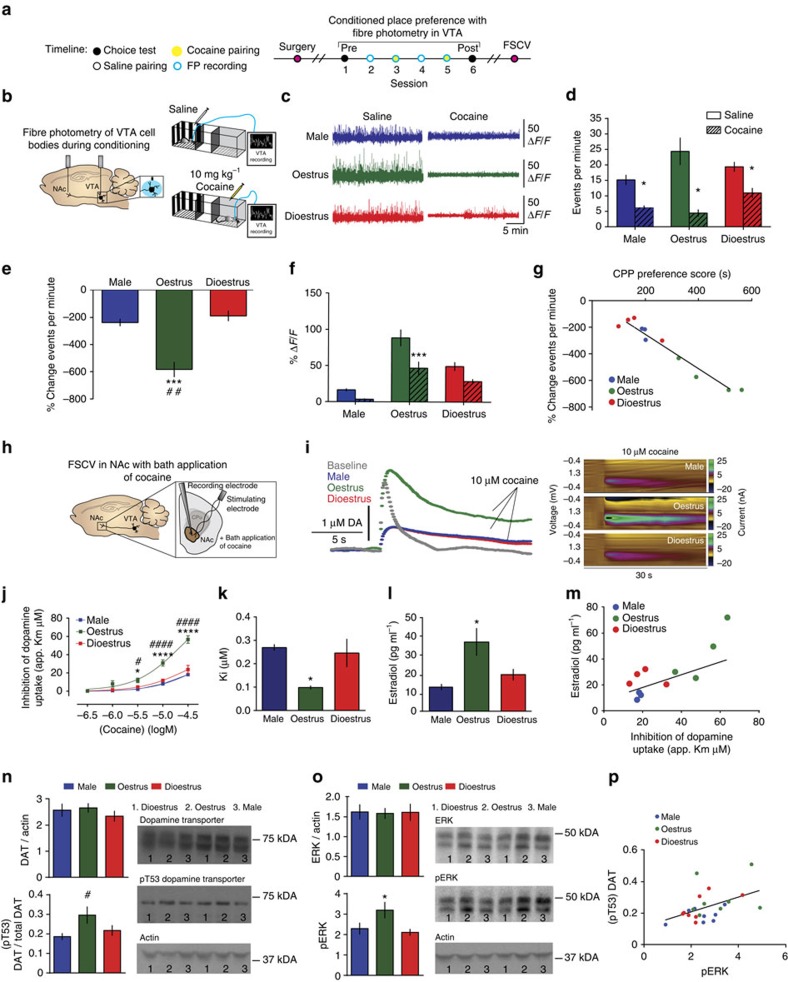
Oestrus females exhibit enhanced cocaine actions on the VTA-NAc reward pathway. (**a**) Timeline of CPP and paired fibre photometry recordings. (**b**) Schematic of fibre photometry recording experiments for VTA recordings during conditioning sessions. (**c**) Representative Ca^2+^ imaging traces from male, oestrus female and dioestrus female mice during saline (left) and cocaine (right) conditioning. (**d**) Cocaine reduces frequency of VTA Ca^2+^ events (two-way analysis of variance (ANOVA); F_(2, 8)_=5.792, *P*<0.05; *P<0.05 as compared with saline). (**e**) Cocaine-induced frequency reductions in activity were greater during oestrus (one-way ANOVA; F_(2, 8)_=23.76, *P*<0.001; ***, *P*<0.001, ^##^*P<*0.01) when compared with males (#) or dioestrus females (*). (**f**) Cocaine-induced changes in the amplitude of Ca^2+^ events (two-way ANOVA; F_(1, 10)_=25.53, *P*<0.001; ***P<0.001 versus saline). (**g**) Correlation between percent change in frequency activity of VTA neurons and CPP (*r*=0.9445; *P*<0.0001). (**h**) Schematic of FSCV recordings with bath application of cocaine performed in NAc slices (right) and representative current versus time plots showing cocaine (10 μM) effects on one pulse evoked dopamine release (left). (**i**) Current versus time plot (left) and colour plots (right) showing the presence of dopamine, as indicated by its oxidation at +0.6 V and reduction at −0.2 V, and the effects of bath application of 10 μM cocaine. (**j**) Concentration–response curves show that cocaine potency is increased selectively in females during oestrus with no difference in baseline activity (two-way ANOVA; F_(4, 16)_=15.73, *P*<0.0001; *P<0.05, ****P<0.0001, ^#^*P<*0.05, ^####^*P*<0.0001). (**k**) *K*_i_ values show that the affinity of cocaine for DAT is increased during oestrus (one-way ANOVA; F_(2, 6)_=6.564, *P*<0.05; *P<0.05). (**l**) Serum estradiol levels were increased during oestrus (one-way ANOVA; F_(2, 13)_=4.83, *P*<0.05; *P<0.05). (**m**) Serum estradiol levels taken immediately before FSCV recordings was positively correlated with cocaine potency (*r*=0.731; *P*<0.01). (**n**) Western blot analysis showing that dopamine transporter levels are not changed over the oestrous cycle (one-way ANOVA F_(2, 21)_=0.68, *P*=0.52) (top), yet levels of the phosphorylated Thr53 site on the dopamine transporter were increased during oestrus (one-way ANOVA F_(2, 21)_=0.37, *P*<0.05; ^#^*P*<0.05) (bottom). (**o**) Total ERK levels were not changed between groups (one-way ANOVA F_(2, 21)_=0.01, *P*=0.99) (top), whereas phosphorylated ERK levels were increased significantly during oestrus (one-way ANOVA F_(2, 21)_=3.97, *P*<0.05; *P<0.05) (bottom). (**p**) These increased phospho ERK levels were correlated with DAT Thr53 phosphorylation (*r*=0.47, *P*<0.05). *P<0.05, **P<0.01, ***P<0.001, ****P<0.0001 oestrus versus dioestrus (unless otherwise noted); ^#^*P<*0.05, ^##^*P<*0.01, ^###^*P<*0.001, ^####^*P<*0.0001 oestrus versus male. Data represented as mean±s.e.m.

Cocaine's ability to alter dopaminergic transmission from the VTA to the NAc has been proposed as a central mechanism underlying its reinforcing and rewarding actions^25^. To examine real-time activity of these neurons *in vivo* during associative learning for drug rewards, we injected male and female C57BL/6J mice with AAV5-CamKII-GCaMP6f into the VTA and recorded Ca^2+^ transients through two separate optic fibres implanted in the VTA and the NAc during cocaine and saline conditioning (Fig. 2a,b and Supplementary Figs 5 and 6a,b)^26^,^27^.

Consistent with electrophysiology data, we show enhanced basal VTA activity in awake and behaving oestrus female mice as compared with both male and dioestrus female mice (Fig. 2c and Supplementary Fig. 7). Further, we show that although cocaine suppressed VTA Ca^2+^ events as measured by both frequency (events per minute) and magnitude (% Δ*F*/*F*) in all groups (Fig. 2c,d,f), the effect of cocaine frequency suppression (% change) in the VTA was significantly higher in oestrus females when compared with male and dioestrus female mice (Fig. 2e). This same reduction in Ca^2+^ activity was also observed at VTA terminals in the NAc, whereby cocaine effects were enhanced selectively during oestrus (Supplementary Fig. 6c–f). Strikingly, the extent to which cocaine suppressed Ca^2+^ events in the VTA cell body and terminal regions was correlated with cocaine CPP in each animal (Fig. 2g and Supplementary Fig. 6g), demonstrating that the increased cocaine effect on the mesolimbic reward system during conditioning is associated with the enhanced reward processing.

### Increased cocaine potency during oestrus

Ultimately, net increases in dopamine in the NAc are proposed to mediate the formation of cue-reward associations^21^. Cocaine elicits elevations in dopamine levels, which leads to decreased VTA and NAc terminal activity via D2 autoreceptor-mediated negative feedback mechanisms^28^. Cocaine's primary molecular mechanism is to increase dopamine concentrations by directly binding to the dopamine transporter (DAT) and inhibiting its ability to clear dopamine from the synaptic cleft^29^,^30^. Using FSCV in NAc slices, we assessed the ability of cocaine to bind to and inhibit DAT in male and female dioestrus and oestrus mice (Fig. 2h). We found that cocaine potency (apparent *K*_m_) is increased in oestrus females (Fig. 2i,j). We also discovered that during oestrus, cocaine's ability to inhibit the clearance of dopamine is potentiated, via a threefold decrease in *K*_i_ (3 × less cocaine is needed to inhibit DAT; Fig. 2k). This result is indicative of an increased affinity for cocaine to bind directly to DAT and inhibit its uptake function in oestrus females.

To assess whether these effects could be due to circulating hormone levels, we assayed progesterone and estradiol levels in serum using enzyme-linked immunosorbent assay (ELISA) within each animal on blood collected immediately before FSCV recordings. We found that circulating estradiol levels were significantly higher in females in oestrus as compared with both dioestrus females and males (Fig. 2l). Further, we found that levels of estradiol, but not progesterone (Supplementary Fig. 8a,b), were predictive of the enhanced cocaine potency at the DAT (Fig. 2m).

Next, we determined whether the effects of estradiol on cocaine potency represented a direct effect at dopamine terminals or an indirect effect via activation of VTA cell bodies. Notably, for all groups, the *K*_m_ (affinity) of dopamine for DAT was not significantly different before cocaine bath application, suggesting that the effects of cocaine on DAT are distinct from any direct effects of estradiol on the transporter (Fig. 2j). We further confirmed this by perfusing NAc slices with estradiol at two different concentrations (1 and 3 μM) and then completing a concentration response curve for cocaine (Supplementary Fig. 9). We found that estradiol had no direct effect on basal DAT function, did not inhibit uptake on its own and did not change the affinity of cocaine for DAT in any of the groups. These findings suggest that the effects of estradiol on the dopamine system originated upstream and lead to downstream adaptations in dopamine terminals via an activity-dependent mechanism.

Although the most parsimonious explanation for these observations is that alterations in cocaine effects on DAT are primarily responsible for changes in cocaine reward learning, it is important to rule out basal differences in dopamine release as a factor driving these effects. Notably, baseline VTA and NAc event frequency did not correlate with the extent of CPP formed within each animal (Supplementary Fig. 10). Moreover, differences in baseline event frequency of VTA neuron cell body and terminals in the NAc did not correlate with cocaine's pharmacological ability to inhibit baseline VTA and NAc event frequency activity (% change in events per minute; Supplementary Fig. 11). In addition, basal dopamine release as measured by voltammetry was not correlated with cocaine's ability to inhibit DAT (Supplementary Fig. 12). Together, these data argue against the possibility that basal differences in increased firing and release drive changes in cocaine reward learning and instead further support our interpretation that oestrous cycle-dependent changes at DAT drive behaviour. Further implicating direct changes to DAT in these processes are data demonstrating that changes in basal uptake function or DAT protein levels are dissociable from differences in cocaine potency (Supplementary Fig. 13; also see ref. 31).

The link between VTA activity and changes at DAT is important, as it is likely to be a compensatory change in response to enhanced dopamine release at the terminal region. Indeed, uptake rates of dopamine via the transporter are increased during oestrus (Supplementary Fig. 13a). Although changes in DAT levels did not explain the changes in uptake rates or cocaine effects during oestrus, we did discover differences in DAT phosphorylation. The DAT is modulated both acutely and chronically by phosphorylation, which can control dopaminergic neurotransmission and signalling quickly and during temporally specific periods^32^. One such site is threonine 53 (Thr53), which is located on the N-terminus of DAT and has been shown to induce conformational changes in the transporter and alter dopamine transport; this change in conformation may be a possible mechanism for increased affinity to stimulants^33^. Our data show that, although levels of DAT are not different among the mice, levels of Thr53 phosphorylation are increased during oestrus (Fig. 2n), which could explain both the enhanced cocaine effects and the increased uptake function. In fact, Thr53 phosphorylation has been linked to both activity-dependent changes in uptake function as well as alterations in stimulant efficacy at the transporter^34^,^35^. Thr53 is phosphorylated by extracellular signal-regulated kinase (ERK) and work has shown that increasing ERK activity leads to Thr53 phosphorylation, enhanced uptake rates and enhanced amphetamine effects at the transporter^34^,^36^,^37^,^38^,^39^,^40^. Similarly, our data show that during oestrus ERK phosphorylation is increased in the NAc (Fig. 2o). The increase in phosphorylated ERK levels was positively correlated with DAT Thr53 phosphorylation (Fig. 2p), suggesting a potential activity-dependent mechanism by which DAT can be modulated during oestrus, to increase the effects of cocaine at DAT and drive enhanced CPP. Together, these data provide a potential oestrous cycle-dependent mechanism for increased cocaine binding to DAT, which is associated with the enhanced behavioural responses to cocaine during the oestrus phase.

### Long-lasting VTA-NAc neural responses to drug-associated cues

**Figure 3:**
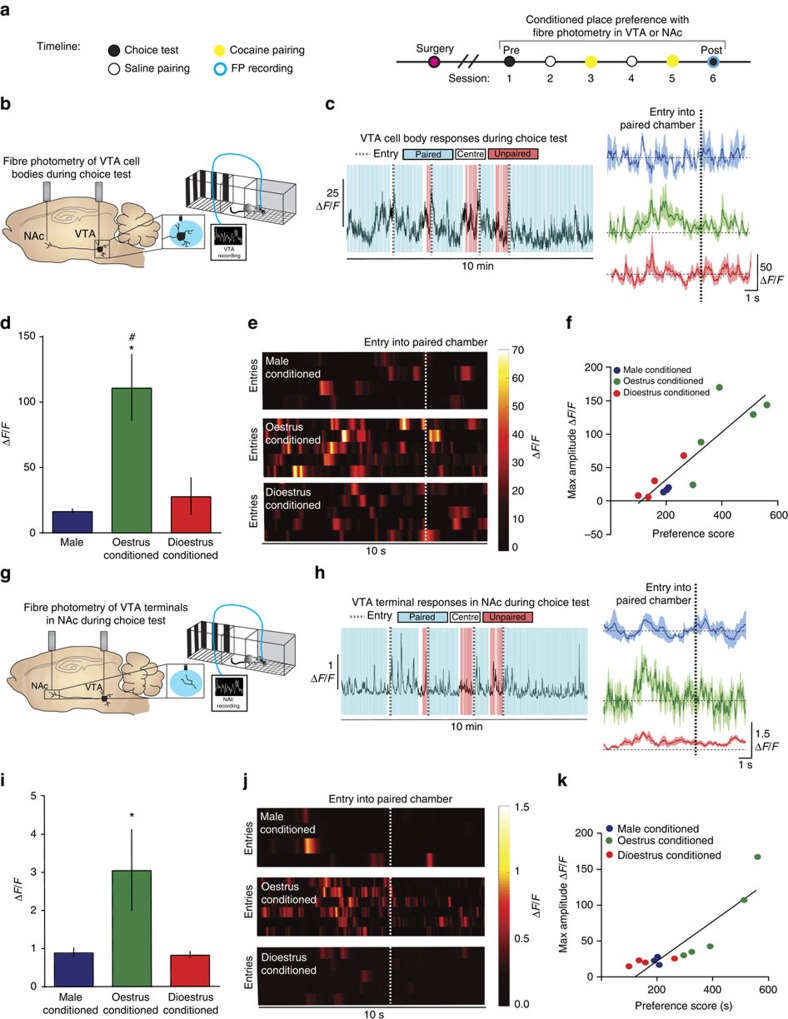
Cocaine-conditioned oestrus females exhibit enhanced VTA and NAc responses to cocaine-associated cues. (**a**) Timeline of CPP experiments and paired fibre photometry recordings during choice test 24 h after the last conditioning session. (**b**) Schematic of recording experiments for VTA cell body recordings, which were taken during the choice test, 24 h after the last conditioning session. (**c**) Representative VTA Ca^2+^ activity trace during choice test demonstrating increases in activity preceding entry (dashed line) into the drug-paired (blue) chamber (left); averaged Ca^2+^ activity in a 10 s window around paired chamber entry (right). (**d**) The amplitude (Δ*F*/*F*) of the spike in Ca^2+^ activity preceding cocaine-paired chamber entry was increased in oestrus-conditioned female mice (one-way analysis of variance (ANOVA); F_(2, 9)_=6.603*, P*<0.05; *P<0.05; ^#^*P*<0.05) when compared with males (#) or dioestrus females (*). (**e**) Heat maps of Ca^2+^ activity over time during successive entries (rows) into the cocaine-paired chamber across groups. (**f**) Correlation between the amplitude of VTA response to the cocaine-paired context and preference score for cocaine (*r*=0.881; *P*<0.0001). (**g**) Schematic of recording experiments for NAc recordings taken at the same time as VTA recordings during the choice test 24 h after the last conditioning session. (**h**) Representative Ca^2+^ activity trace, from the same animal as depicted in **c**, showing VTA terminal activity in the NAc around cocaine-paired (blue) and unpaired (red) entry where dashed lines denote entry into the cocaine-paired chamber (left); averaged Ca^2+^ activity in a 10 s window around paired chamber entry (right). (**i**) The amplitude of the spike in VTA terminal Ca^2+^ activity in NAc preceding cocaine-paired chamber entry was increased in oestrus-conditioned female mice (Kruskal–Wallis (KW); *χ*^2^_(2)_=8.122, *P*<0.01; *P<0.05). (**j**) Heat maps of Ca^2+^ activity over time during successive entries (rows) into the cocaine-paired chamber across groups. (**k**) Correlation between the amplitude of VTA terminal Ca^2+^ activity in NAc to the cocaine-paired context and CPP for cocaine (*r*=0.883; *P*<0.0001). *P<0.05 versus dioestrus; ^#^*P*<0.05 versus male. Data represented as mean±s.e.m.

The VTA-NAc pathway is a key neural substrate of cue-reward associations, as it mediates multiple aspects of this process including learning, selecting and executing goal-oriented behaviours^41^. Over time, contextual cues in the absence of drug can elicit neural responses that resemble that of the drug itself^23^,^42^ and trigger drug craving and seeking, resulting in relapse in drug-addicted individuals^43^,^44^,^45^,^46^,^47^. The data presented thus far define one mechanism for the enhanced rewarding effects of cocaine during oestrus; however, they do not explain the enhanced cue-induced drug-seeking and relapse that occurs in females when drugs of abuse are no longer on board. Using *in vivo* fibre photometry Ca^2+^ imaging, we recorded from VTA cell bodies and their terminals in the NAc during the choice test of the CPP paradigm of male mice and female mice that were conditioned during proestrus/oestrus or dioestrus (Fig. 3a,b). We then analysed neural activity when mice entered the cocaine-paired context or the saline-paired context.

We found that increases in VTA Ca^2+^ activity preceded entry into the cocaine-paired, but not saline-paired, chamber (Fig. 3c,e and Supplementary Fig. 14). Interestingly, the magnitude of these responses (Δ*F*/*F*) was significantly higher in oestrus-conditioned female mice (Fig. 3c–e). It is important to note that these females are no longer in oestrus during the choice testing; thus, the associations that are formed between drugs and associated cues during oestrus are potent and long lasting. Furthermore, the amplitude of these signals was correlated significantly with the extent of place preference, where the mice with the largest amplitude of VTA response to the contextual cues exhibited the greatest CPP (Fig. 3f). Equivalent responses were seen for VTA nerve terminals in the NAc, whereby Ca^2+^ transients preceded drug paired, but not unpaired, entry (Fig. 3g,h). Accordingly, the Ca^2+^ transients in NAc that preceded drug-paired entry correlated with VTA activity at the same time point (Fig. 3c,h), were increased in oestrus-conditioned females (Fig. 3i,j) and were correlated with the extent of the conditioned preference (Fig. 3k). These data show that temporally precise signalling in the VTA-NAc pathway encodes cocaine-context associations, which act to enhance choice for drug-associated contexts.

**Figure 4:**
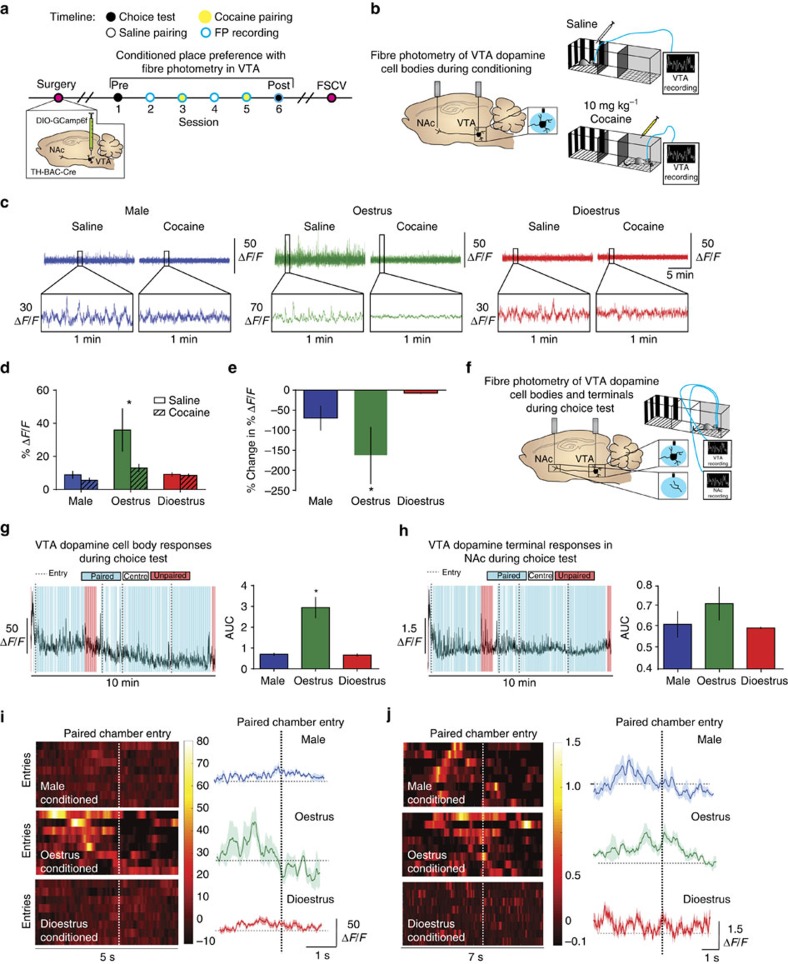
Oestrous cycle-dependent fluctuations in VTA and NAc are mediated by dopaminergic signalling. (**a**) Timeline of CPP experiments and paired fibre photometry recordings during choice test 24 h after the last conditioning session. (**b**) Schematic of recording experiments for VTA cell body recordings in *TH-BAC-Cre* mice, which were taken during the saline or cocaine pairing. (**c**) Representative Ca^2+^ imaging traces from male (left), oestrus female (middle) and dioestrus female (right) mice during saline and cocaine conditioning. (**d**) Cocaine reduces the amplitude of VTA Ca^2+^ events (two-way analysis of variance (ANOVA); F_(1, 8)_=8.63, *P*<0.01; *P<0.05 as compared with saline). (**e**) Cocaine-induced amplitude reductions in activity were greater during oestrus (one-way ANOVA; F_(2, 8)_=23.76; *P<0.05) when compared with males or dioestrus females. (**f**) Schematic of recording experiments for VTA cell body and terminal recordings in *TH-BAC-Cre* mice, which were taken during the choice test, 24 h after the last conditioning session. (**g**) Left: representative VTA Ca^2+^ activity trace during choice test demonstrating increases in activity preceding entry (dashed line) into the drug-paired (blue). (**g**) Right: the area under the curve (Δ*F*/*F*) of the spike in Ca^2+^ activity preceding cocaine-paired chamber entry was increased in oestrus-conditioned female mice (one-way ANOVA; F_(2, 8)_=5.80, *P*<0.05; *P<0.05). (**h**) Left: representative NAc Ca^2+^ activity trace. (**h**) Right: the area under the curve (Δ*F*/*F*) of the spike in Ca^2+^ activity in NAc terminals preceding cocaine-paired chamber entry (one-way ANOVA; F_(2, 8)_=4.50, *P*<0.05). (**i**) Left: heat maps of VTA-dopamine Ca^2+^ activity over time during successive entries (rows) into the cocaine-paired chamber across groups. (**i**) Right: averaged VTA-dopamine Ca^2+^ activity in a window around paired chamber entry. (**j**) Left: heat maps of NAc terminal-dopamine Ca^2+^ activity over time during successive entries. (**j**) Right: averaged NAc terminal-dopamine Ca^2+^ activity in a window around paired chamber entry. Data represented as mean±s.e.m.

Although the data presented above outline a role for the VTA-NAc pathway in this process, they do not rule out the contribution of non-dopaminergic projections originating in the VTA in encoding this information. To this end, we used male and female mice that express Cre-recombinase under the control of the TH promoter (*TH-BAC-Cre*), combined with viral expression of Cre-dependent AAV5-DIO-GCaMP6f in the VTA, to record dopamine neuron-specific Ca^2+^ transients through two separate optic fibres implanted in the VTA and the NAc during cocaine and saline conditioning (Fig. 4a,b)^26^,^27^,^48^. Consistent with electrophysiology data and the global imaging data, we show enhanced basal VTA activity of dopaminergic neurons in awake, behaving oestrus female mice as compared with both male and dioestrus female mice (Fig. 4c,d and Supplementary Fig. 15). Further, we show that, although cocaine suppressed VTA neural activity (Fig. 2c,d), the effect of cocaine in the VTA was significantly higher in oestrus females when compared with male and dioestrus female mice (Fig. 4c–e).

In addition, we recorded from dopamine neuron cell bodies in the VTA and their terminals in the NAc during the choice test and then analysed the Ca^2+^ activity that occurred when mice entered the cocaine-paired context (Fig. 4f). Again, the magnitude of these responses (Δ*F*/*F*) was significantly higher in oestrus-conditioned female mice when recording specifically from VTA dopamine cell bodies (Fig. 4g,i). Equivalent responses were seen for VTA dopamine nerve terminals in the NAc, whereby Ca^2+^ transients preceded drug-paired entry (Fig. 4h,j). These data provide confirmation that temporally precise dopamine signalling in the VTA-NAc pathway encodes cocaine-context associations.

### VTA dopamine neuron firing controls DAT function

**Figure 5:**
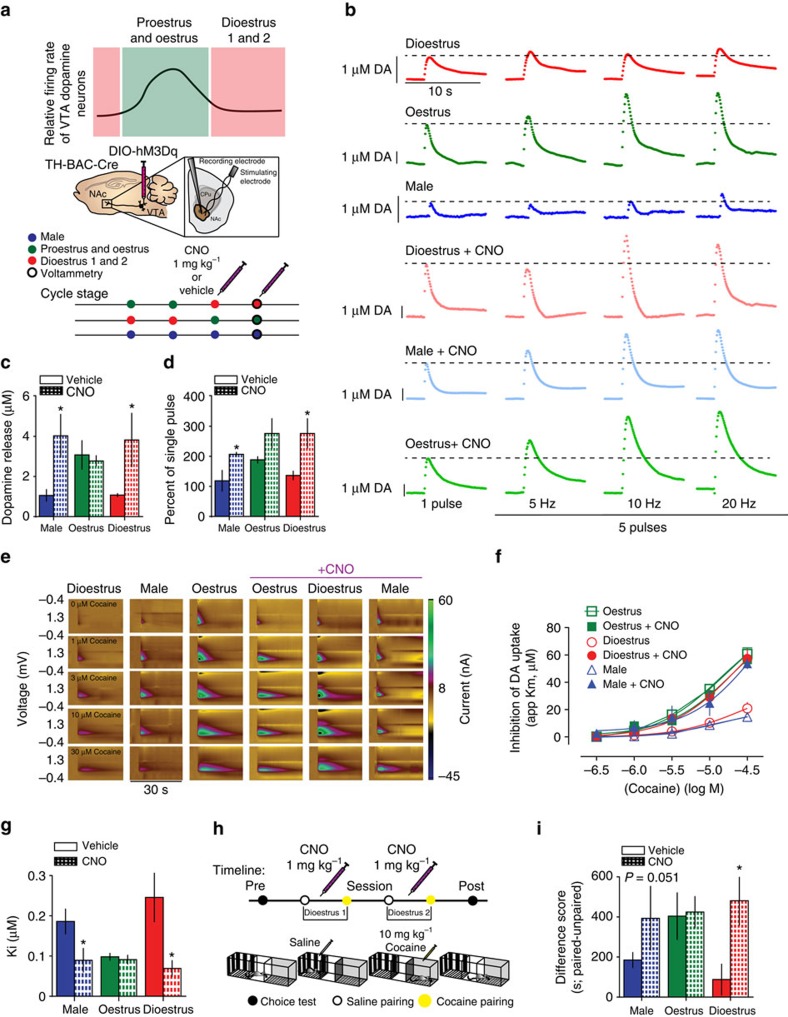
Enhancing VTA dopamine activity in dioestrus females increases cocaine reward processing. (**a**) Schematic of VTA firing through the oestrous cycle (top); experimental design using excitatory DREADDS (hM3Dq) expressed exclusively in dopamine neurons using *TH-BAC-Cre* mice to increase VTA dopamine activity (bottom). (**b**) Representative FSCV current versus time plots showing dopamine release to increasing frequency stimulations during oestrus, dioestrus or in males on the second day of vehicle or CNO injections. (**c**) Group data demonstrate enhanced dopamine per stimulation in dioestrus females and males that were given CNO to enhance VTA firing rates (one-way analysis of variance (ANOVA); F_(5, 12)_=3.78, *P*<0.05; *P<0.05). (**d**) Phasic responsivity was increased in dioestrus females and males given CNO (two-way ANOVA; F_(5, 8)_=3.93, *P*<0.05; *P<0.05. (**e**) Colour plots showing the presence of dopamine, as indicated by its oxidation at +0.6 V and reduction at −0.2 V, and the effects of bath application of 1, 3, 10 and 30 μM cocaine in each group. (**f**) Concentration–response curves showing that cocaine potency is increased in oestrus females and dioestrus females that were given CNO as compared with dioestrus controls. (**g**) *K*_i_ values showing that the affinity of cocaine for DAT is increased in dioestrus females and males given CNO (one-way ANOVA; F_(5, 12)_=3.89, *P*<0.05; *P<0.05). (**h**) Schematic of CPP experiments with CNO injections. (**i**) Increased CPP in male and dioestrus females with CNO+DREADDs to increase VTA firing (one-way ANOVA; F_(5, 12)_=3.78, *P*<0.05; *P<0.05 versus dioestrus). Data represented as mean±s.e.m.

We show that oestrous cycle-dependent changes in reward system function at the level of both the VTAs and terminals in the NAc lead to fluctuations in the ability to associate rewards with contextual cues; however, the mechanism was unclear. We hypothesized that this enhancement of VTA firing was the mechanism by which DAT and dopamine release was altered to enhance cocaine effects and concomitant reward processing. Using male and female *TH-BAC-Cre* mice combined with viral expression of Cre-dependent excitatory designer receptors exclusively activated by designer drugs (excitatory DREADDs: hM3Dq) we were able to use clozapine-N-oxide (CNO) to enhance VTA dopamine neuron firing selectively in males and dioestrus females (Fig. 5a)^48^,^49^. We find that oestrus females, compared with dioestrus females and males release two times more dopamine to single-pulse tonic stimulations in the NAc (Fig. 5b,c). However, when VTA dopamine neuron firing is chemogenetically increased specifically during dioestrus, dopamine release was increased compared with control dioestrus females and males, and not different from oestrus females (Fig. 5b,c). Furthermore, enhancing basal VTA firing is sufficient to increase male and dioestrus female's responsivity to phasic stimulation parameters to match those of oestrus females (Fig. 5b,d).

The ability of cocaine to inhibit DAT (apparent *K*_m_) and elevate dopamine levels is increased in oestrus females (Fig. 5e–g). When excitatory DREADDs are used to enhance selectively VTA dopamine neuron firing in dioestrus females and males, the ability of cocaine to inhibit DAT is increased to the levels of oestrus females (Fig. 5f,g). CNO alone in mice that did not express DREADDs did not affect any of these measures, further highlighting that the effects were due to DREADD-induced increases in VTA activity (Supplementary Fig. 16). This is concomitant with a threefold increase in the affinity for cocaine to bind to DAT (Fig. 4g). These data demonstrate that enhancement of VTA firing is sufficient to induce changes in protein function that alter substrate binding. In addition, cocaine-induced dopamine release is increased in oestrus females. Thus, increasing VTA firing during dioestrus recapitulates both the basal dopamine function and effects of cocaine seen during oestrus.

**Figure 6:**
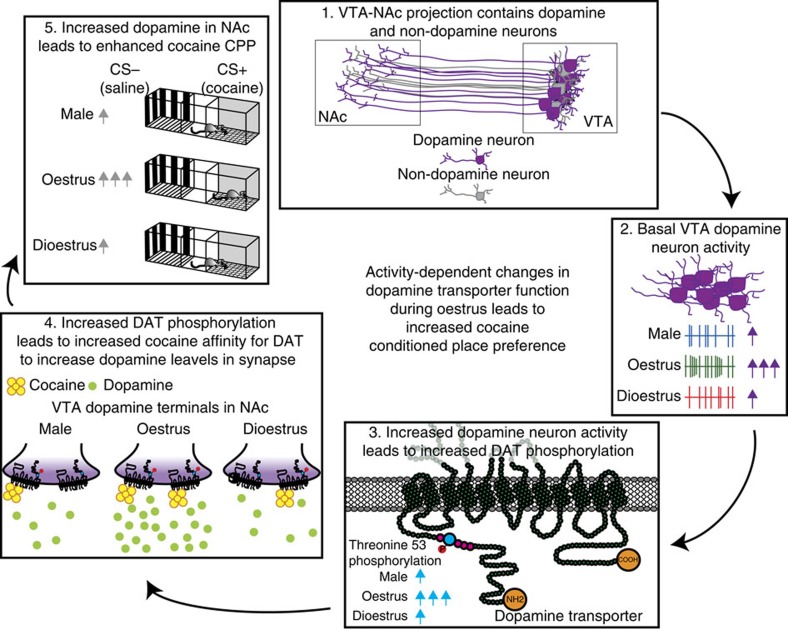
Proposed schematic highlighting a potential mechanism for the activity-dependent changes in reward processing that occur during oestrus. (1) The VTA to NAc pathway comprises dopaminergic neurons (purple) and other neuronal subpopulations (grey). (2) Dopamine neuron firing is enhanced during oestrus. (3) The increased activity of this pathway leads to downstream ERK activation and concomitant phosphorylation of Thr53 (blue) on DAT. (4) These changes in DAT lead to alterations in cocaine affinity, whereby cocaine is more able to bind to DAT and increase extracellular dopamine levels. This increased cocaine binding leads to increased dopamine levels in the NAc. (5) *In vivo* this drives increased associations between cocaine and contextual cues, which leads to enhanced cocaine CPP due to differences in the perceived rewarding value of cocaine.

To determine whether the increased VTA dopamine activity associated with the oestrus cycle is causal to enhanced reward processing, we increased VTA dopamine activity in *TH-BAC-Cre* male, dioestrus female and oestrus female mice during cocaine conditioning using excitatory DREADDs (Fig. 5h). The formation of CPP in CNO-treated dioestrus females and males is increased to levels similar of oestrus females (Fig. 5i). However, when this same experiment was done in oestrus females, CNO was not able to further potentiate cocaine CPP. This provides additional support for the idea that downstream changes in reward processing and CPP are mediated by activity-dependent alterations in dopaminergic projections from the VTA to the NAc (Fig. 6).

## Discussion

We define a mechanism by which a selective increase in drug potency during oestrus leads to long-lasting associations that enhance drug responses in female mice even outside of the oestrus phase. First, we identify a mechanism for the enhanced cocaine reward exhibited in oestrus females by showing that cocaine affinity for DAT is enhanced during oestrus. Levels of circulating estradiol were correlated with cocaine potency highlighting its role in the process. This enhances dopaminergic responses to both cocaine and reward cues, leading to VTA-NAc responses to the environmental cues alone, in the absence of drug, even at later stages in the oestrous cycle. Second, we determined the mechanism by which the DAT is altered to have a higher affinity for cocaine binding. Oestrous cycle-dependent enhancement of VTA firing leads to activity-dependent changes at the level of DAT that enhanced cocaine potency and subsequent cue-reward associations for cocaine. This enhanced dopaminergic function paired with enhanced cocaine action directly at its site of action lead to potent and long-lasting associations between drugs and associated cues that likely enhance motivation, drug-seeking and relapse, even in the absence of drug. Together, these data provide a basic mechanism whereby increased VTA firing drives adaptations to DAT that increase cocaine potency at DAT and increase cocaine reward.

Previous work using anaesthetized *in vivo* voltammetry and microdialysis has shown that cocaine increases extracellular dopamine levels to a greater extent in oestrus females as compared with females in dioestrus; however, the mechanism for this effect was unclear and was suggested to be mediated selectively via effects on dopamine release^50^. Here we show that these increases in cocaine's ability to increase dopamine levels in reward-related brain regions are due to an increase in cocaine's ability to bind to and inhibit DAT. Although dopamine release was also increased, a finding that is consistent with a large body of previous work, we found that the enhanced release was not predictive of changes in cocaine's ability to inhibit uptake; thus, the two phenomena were dissociable. Further, the magnitude of cocaine's effects on the dopamine system was predictive of CPP, showing that this enhanced ability to inhibit DAT probably leads to increased associations between the rewarding properties of cocaine and the contextual cues that predicted its availability. These data could also explain the increased acquisition and drug consumption that is seen during oestrus in animals voluntarily self-administering cocaine^51^,^52^.

As the effects we observed were oestrous cycle dependent, we hypothesized that they were driven by circulating levels of ovarian hormones. To this end, we determined serum levels of estradiol and progesterone within each subject and found that the increase in cocaine potency at DAT was highly correlated with estradiol, but not progesterone levels. Previous work in ovariectomized rodents has shown that systemic delivery of estradiol, but not progesterone, increases cocaine consumption and motivation, suggesting that these changes in cocaine potency could be the molecular mechanism by which these behavioural effects occur^53^,^54^. Further, we determined where these hormone-induced changes in cocaine potency on dopamine terminals originated. We bath applied estradiol directly to slices containing the NAc. These coronal slices contained dopamine nerve terminals originating in the VTA, but disconnected from their associated VTA cell bodies. We found that estradiol did not affect baseline dopamine nerve terminal function or cocaine potency in any of the groups tested, suggesting that the changes in the function of the dopamine terminal originated upstream.

The hypothesis that upstream changes at the level of the cell body in the VTA drive this process was supported by data showing that enhancement of VTA activity via DREADDs was able to change the dopamine terminal and enhance cocaine reward in male and dioestrus female mice. Thus, together we hypothesize that during oestrus increases in VTA firing lead to downstream, activity-dependent signalling that alters DAT. Increasing VTA dopamine activity via excitatory DREADDs increased both the affinity of cocaine for DAT and the formation of CPP in males and dioestrus females. We found that these changes in cocaine effects at DAT were concomitant with increased DAT phosphorylation at Thr53. In fact, Thr53 phosphorylation has been linked to both activity-dependent changes in uptake function and alterations in stimulant efficacy at the transporter^34^. Increasing ERK activity leads to Thr53 phosphorylation, enhanced uptake rates and enhanced stimulant effects at the DAT^36^,^37^,^38^,^39^,^40^. Further, previous work has shown that cellular depolarization and activity is sufficient to increase ERK activity^55^, providing a link between enhanced DAT phosphorylation, and increases neuronal activity. Indeed, here we show that during oestrus phosphorylated ERK levels are increased, and that this increase is directly correlated with DAT phosphorylation at Thr53. Further work is needed to establish causal connections between Thr53 phosphorylation and oestrous cycle-dependent fluctuations in reward processing. One important finding to support this hypothesis was the lack of further increases in cocaine potency in oestrus females expressing excitatory DREADDs. The occlusion of activity-dependent changes in cocaine effects was observed not only at the level of the dopamine terminal, but also in regard to the development of CPP. Although this is not definitive proof, this occlusion strongly suggests that these two effects are probably occurring through similar molecular mechanisms.

Further, these data are important, because these molecular adaptations drive strong cue-reward associations that extend past the oestrus stage. Thus, it seems that when the cues are associated may play a large role in determining the subsequent response to cues. The ability to associate rewards with cues that predict their availability is critical to survival, as these cues can result in the motivation to obtain rewards such as food and underlie the motivation to reproduce^41^. However, drugs of abuse hijack natural reward-learning processes, with associative learning contributing not only to the increased motivation to administer drugs of abuse such as cocaine, but also to relapse in both humans and animal models^44^,^45^. Greater cocaine effects on this pathway are associated with the compulsive drug-seeking and taking that develops over time and characterizes an addicted state^56^. Results of the present study show that oestrous cycle-dependent enhancement of reward circuitry and cocaine effects directly on this system lead to potent and long-lasting associations between the rewarding effects of cocaine and the environmental cues that predict its availability and the activity-dependent mechanism by which this occurs.

Together, we propose a mechanism by which oestrous cycle-dependent fluctuations in estradiol lead to increases in VTA activity, which increases ERK activity and leads to conformational changes in DAT via Thr53 phosphorylation (Fig. 6). These changes in DAT enhance cocaine's ability to inhibit its uptake function and augment cocaine-induced elevations in dopamine levels. In turn, these enhanced dopaminergic responses increase the rewarding properties of cocaine and enhance the cue-reward associations that are formed as a result. These associations are strong enough that the cues, in the absence of cocaine, elicit dopaminergic responses and drive animals to seek the reward (that is, cocaine) that was previously associated with them. These associations, which are stronger in females during oestrus, could lead to the precipitation of relapse even after periods of abstinence.

More broadly, we have defined a basic mechanism whereby enhanced activity of a pathway can lead to alterations in protein function that enhance reward processing. In humans, people with anxiety and depression are two times more likely to have problems with drugs of abuse. Accordingly, previous work in animals has shown that mice that are susceptible to developing depressive-like behaviours have enhanced VTA excitability and firing rates, as well as increased responses to stimulant drugs of abuse. Thus, this work has implications for not only drug addiction but also a number of psychiatric disorders and may underlie the comorbidity of disorders such as anxiety and depression with drug addiction. Taken together, our findings reveal novel sex-specific factors controlling reward processing and are critical to guiding evidence-based therapeutic interventions for females.

## Methods

### Animals

Male and female 6- to 8-week-old C57BL/6J and *TH-BAC-Cre* mice were maintained on a 12 h light–dark cycle at 22–25 °C with *ad libitum* access to food and water. C57BL/6J wild-type mice were obtained from Jackson Laboratories (Bar Harbor, MN; SN: 000664). *TH-BAC-Cre* mice from GENSTAT were bred and maintained by the Center for Comparative Medicine and Science staff at Icahn School of Medicine at Mount Sinai Mice until transfer for experimental use. All mice were housed three to five per cage. Female mice were subjected to daily vaginal smears and vaginal cytology was conducted to determine oestrous cycle phase. All experiments were conducted in accordance with the guidelines of the Institutional Animal Care and Use Committee at Icahn School of Medicine at Mount Sinai, which approved and supervised all animal protocols. Experimenters were blind to experimental group and order of testing was counterbalanced during behavioural experiments.

### Drugs

CNO (Sigma-Aldrich) was dissolved at a concentration of 2.5 mg ml^−1^ in 5% dimethyl sulfoxide in normal saline (Sigma-Aldrich). CNO was administered in the following dose of 1 mg kg^−1^, i.p. An equal (matched) volume of vehicle (saline with 5% dimethyl sulfoxide) was used as the control. Cocaine HCl was provided by the NIDA drug supply programme and administered at a 10 mg kg^−1^ doses i.p. For slice voltammetry experiments, Cocaine (NIDA Drug Supply), Quinpirole (Sigma-Aldrich) and Estradiol (Sigma-Aldrich) were perfused over slices in artificial cerebrospinal fluid (aCSF) solution.

### Viral constructs

For general GCaMP expression in VTA neuronal populations, GCaMP6f was cloned into the adeno-associated virus (AAV) vector, AAVdj-CaMKIIα-GCaMP6f-WPRE. For dopamine-specific GCaMP expression in TH(+) populations of the VTA, cre-inducible (DIO) GCaMP6f was cloned into the AAV vector, AAVdj-DIO-EF1α-GCaMP6f-WPRE, and injected into the VTA of *TH-BAC-Cre* mice. For DREADD experiments in dopamine populations of the VTA, *TH-BAC-Cre* animals were injected with AAV2-hSyn-DIO-hM3Dq-mCherry that was purchased from the viral core at the University of North Carolina, Chapel Hill.

### Stereotaxic virus injection and optic fiber implantation

Under ketamine (100 mg kg^−1^)/xylazine (10 mg kg^−1^) anaesthesia, mice were positioned in a stereotaxic frame (Kopf Instruments) and the VTA was targeted (Bregma coordinates: anterior/posterior, −3.3 mm; medial/lateral, 0.75 mm; dorsal/ventral, −4.6 mm; 7° angle). Ophthalmic ointment was applied to the eyes to prevent drying. A midline incision was made down the scalp and a craniotomy was made using a dental drill. A 10 μl Nanofil Hamilton syringe (WPI, Sarasota, FL) with a 34-gauge beveled metal needle was used to infuse 0.5 μl virus at a rate of 100 nl min^−1^. Following infusion, the needle was kept at the injection site for 10 min and then slowly withdrawn. Chronically implantable optic fibres constructed with 400 μm core 0.48 numerical aperture (NA) optic fibre and unilaterally implanted into the VTA (Bregma coordinates: anterior/posterior, −3.3 medial/lateral, +1.0; dorsal/ventral, −4.4; 7° angle) and NAc (bregma coordinates: anterior/posterior, 1.4 mm; medial/lateral, 1.5 mm; dorsal/ventral, −4.3 mm; 10° angle), and were cemented to the skull using dental acrylic (Grip Cement Dentsply). For robust viral expression, viral infusions and implantation occurred a minimum of 4–6 weeks before recordings.

### Conditioned place preference

Daily oestrous cycle staging was conducted 1.5 h before experiments. All mice were acclimated to the behaviour room for at least 1 h before testing. CPP was performed in a rectangular apparatus consisting of two side chambers measuring 28 × 24 cm each and a centre chamber measuring 11.5 × 24 cm. One side had black and white stripes on the walls with a metal grid floor and the other had grey walls with a punched metal floor. An unbiased CPP paradigm was used in the current study. On Day 1, mice were allowed to explore the entire apparatus for 20 min. On days 2 and 3, mice were confined to one of the side chambers and injected with cocaine or saline. Side pairings were counterbalanced across animals. On Day 5, during the choice test, mice were again allowed to freely explore the entire apparatus. Preference was determined by a difference score (time spent in paired−time spent in unpaired chamber). For calcium imaging during conditioning or during choice tests, a 3 m-long fibre-optic patch-cord (Doric Lenses) was connected to the chronically implanted optical fiber with a zirconia sheath and was suspended above the behavioural testing environment, to allow animals to move freely during stimulation. Noldus software running Ethovision 10.0 recorded behavioural outputs.

### CPP with DREADD-induced excitation of VTA dopamine neurons

To determine whether basal increases in VTA firing were sufficient to enhance reward processing, AAV-DIO-hM3Dq was injected into the VTA of *TH-BAC-Cre* mice. The virus was allowed to express for 5 weeks and then the animals went through CPP as described above. CNO (1 mg kg^−1^; Sigma) was administered 2 h before cocaine pairing sessions. Time spent in each area of the conditioning chamber was recorded as before and animals were considered to have developed a preference when the difference in time spent between the drug paired and chamber paired with saline was significantly greater than before the pairings.

### *In vivo* anaesthetized single-unit recordings

*In vivo* extracellular, single-unit recordings to determine basal activity of VTA dopamine neurons of anaesthetized intact mice were conducted following published methods^57^. Male and oestrus and dioestrus female mice were anaesthetized with 8% chloral hydrate (400 mg kg^−1^, i.p.) and head fixed to a stereotaxic frame. The skull was exposed to identify bregma and coordinates were measured to determine VTA location where a skull window was created (anterior/posterior, −2.92∼−3.88; medial/lateral ±0.25∼0.96; and DV=−3.5∼−4.5). Extracellular single-unit signals were amplified using a DP-311 Differential Amplifier and filtered (0.3–1 kHz band pass). Voltage data were acquired using an Axon Digidata Data Acquisition System, sampled using 16-bit resolution at 32 kHz and stored using pCLAMP for further offline analysis.

### Single-unit recordings data analysis

Putative dopamine cells in the VTA were identified according to standard electrophysiological criteria: a stark, triphasic waveform with set filters; action potential duration from start to negative trough ≥1.1 ms; slow firing rate (<10 spikes per second); and bursting behaviour with onset beginning when two spikes occurred within <80 ms and offset set when no activity occurred for ≥160 ms^58^. Spike times were measured using Clampfit 10.2 and data were further processed using *R* as previously described^59^. Activity was quantified by overlapping 60 s windows shifted every 15 s. Percentage of spikes within bursts corresponds to the percentage of spikes discharged within a burst. Further, burst length was determined after measuring the number of spikes within a burst and averaged per cell.

### Fast-scan cyclic voltammetry

*Ex vivo* FSCV was used to characterize D2/D3 autoreceptor function, DAT activity, dopamine release and cocaine potency in the NAc. Voltammetry experiments were conducted 1–2 weeks following the completion of the CPP experiments. Recordings were made directly at the end of the fiberoptic probe, to ensure that the recordings were from the same location in the NAc as calcium-imaging experiments. A vibrating tissue slicer was used to prepare 250 μm-thick coronal brain sections containing the NAc, which were immersed in oxygenated aCSF containing (in mM): NaCl (126), KCl (2.5), NaH_2_PO_4_ (1.2), CaCl_2_ (2.4), MgCl_2_ (1.2), NaHCO_3_ (25), glucose (11), L-ascorbic acid (0.4) and pH was adjusted to 7.4. The slice was transferred to the testing chambers containing aCSF at 32 °C with a 1 ml min^−1^ flow rate. A carbon fibre microelectrode (100–200 μM length, 7 μM radius) and bipolar stimulating electrode were placed into the NAc where GCaMP-GFP terminal expression was high and near the optic fibre track to get the best representation of release near the recorded regions. Dopamine release was evoked by a single electrical pulse (300 μA, 4 ms, monophasic) applied to the tissue every 5 min. The extracellular dopamine level was recorded by applying a triangular waveform (−0.4 to +1.2, to −0.4 V versus Ag/AgCl, 400 μV s^−1^). Once the peak of evoked dopamine release was stabilized (three collections with <10% variability), the amount of evoked dopamine release and a maximal rate of uptake (*V*_max_) were assessed. Subsequently, tonic and phasic stimulations were applied to slices, to determine the responsivity of VTA terminals in the NAc to tonic and phasic firing parameters. Tonic stimulations consisted of one pulse stimulations. Phasic stimulations consisted of five pulses at either 5, 10 or 20 Hertz. These stimulation parameters were selected based on the physiological firing properties of VTA dopamine neurons *in vivo*.

To assess autoreceptor function, quinpirole, a D2/D3 receptor agonist, was applied cumulatively to the brain slice, to determine a concentration–response curve (0.01–1 μM) to assess quinpirole effects on dopamine release. In a slice preparation, presynaptic autoreceptors are dissociated from postsynaptic D2 receptors. As quinpirole activates autoreceptors, evoked dopamine release is reduced. The resulting concentration–response curve is then fit to determine the IC50 of quinpirole in each condition. Increases in autoreceptor sensitivity increase the IC50 of qunipirole on dopamine release.

To determine cocaine potency, cocaine was applied cumulatively to brain slices. Once the extracellular dopamine response was stable, cocaine (0.03–30 μmol l^−1^) was applied and resulting current versus time plots were analysed at each concentration, to determine the ability of cocaine to inhibit DAT and elevate synaptic dopamine levels.

To determine the local effects of estradiol on NAc brain slices, estradiol was bath applied at 1 or 3 μM. Recordings were taken for 60 min at each dose, to ensure that estradiol had sufficient time to induce changes. Once dopamine measurements were stable, a concentration response curve for cocaine was run (0.1–30 μmol l^−1^) within each group.

Recording electrodes were calibrated by recording responses (in electrical current; nA) to a known concentration of dopamine (3 μM) using a flow-injection system. This was used to convert an electrical current to a dopamine concentration.

### Voltammetric data analysis

Demon voltammetry and analysis software was used for all analysis of FSCV data^60^. Data were modelled using Michaelis–Menten kinetics to determine dopamine release, *V*_max_ and cocaine potency. The quinpirole concentration–response curves were assessed by determining the peak height of dopamine release at each dose and expressed as a percentage of the pre-drug basal dopamine release.

To evaluate dopamine kinetics and drug potency, evoked levels of dopamine were modelled using Michaelis–Menten kinetics. Apparent *K*_m_ (App. *K*_m_), a measure of the ability of a drug to inhibit dopamine clearance, was used to evaluate changes in cocaine potency. App. *K*_m_ was determined from the decreased affinity of dopamine (*K*_m_) for the DAT. As the ability of DAT to clear dopamine is decreased (following application of cocaine), the extent to which this occurs can be calculated. The decreased affinity of dopamine (*K*_m_) for DAT is then used to determine an App. *K*_m_ of the drug for the transporter. For cocaine concentration–response curves, App. *K*_m_, a measure of apparent affinity for the DAT, was used to determine changes in ability of the cocaine to inhibit dopamine uptake. As App. *K*_m_ increases, uptake inhibition increases and changes in drug potency are seen as shifts in the curve.

Inhibition constants (*K*_i_) were determined by plotting the linear concentration-effect profiles and determining the slope of the linear regression. The *K*_i_ was calculated by the equation *K*_m_/slope. *K*_i_ values are reported in μM and are a measure of the drug concentration that is necessary to produce 50% uptake inhibition. *K*_i_ is a measure of the actual drug affinity for the DAT in concentration of drug, whereas *K*_m_ is a dopamine-related parameter that estimates uptake inhibition.

### Blood collection

Trunk blood was collected in LoBind tubes on day of FSCV recordings. Blood was allowed to clot at room temperature for one hour and centrifuged at 2,500 r.c.f. at 4 °C, to separate serum and erythrocytes. Serum was stored at −20 °C until day of analysis.

### Enzyme-linked immunosorbent assay

ELISA kits for estradiol and progesterone (Cayman Chemical) were used to measure serum samples from oestrus and dioestrus females and males. Blood samples were taken immediately before FSCV recordings. The appropriate estradiol or progesterone standards of the manufacturer, and negative and positive controls were run on each plate. A 10 μl aliquot of diluted samples was pipetted manually into the plate wells in duplicate. The ELISA assays were then run according to the instructions of the manufacturers. The samples were incubated in microplate wells coated with fixed amounts of oriented high-affinity purified polyclonal antibody and the mixture was incubated at room temperature for 60 min in the dark. After incubation, the plate was washed six times with deionized H_2_O, to remove any unbound sample or drug–enzyme conjugate. The chromogenic substrate was then added (100 μl per well) and the plate was incubated for 30 min in the dark. After substrate incubation, the reaction was halted with the addition of an acid-based stop solution (100 μl per well). The plate was read using a Spectra max plus plate reader equipped with a 450 nm filter (Molecular Devices).

### FSCV with DREADD-induced excitation of VTA dopamine neurons

To determine whether basal increases in VTA firing were sufficient to alter basal dopamine system function, AAV-DIO-hM3Dq-mCherry was injected into the VTA of male and female *TH-BAC-Cre* mice. The virus was allowed to express for 5 weeks. Vaginal cytology was done starting 2 weeks before the voltammetry experiments, to determine the cycle stage of the animals. For all groups DREADDs were expressed in VTA dopamine neurons and activated with CNO (1 mg kg^−1^). Vaginal cytology was done before FSCV recordings, to confirm cycle stage on day of recording.

### Fibre photometry calcium imaging

Fibre photometry uses the same optic fibre to both excite and record from the genetically encoded calcium indicator GCaMP6f in real time and thus can allow for temporally precise measurements of neuronal activity that can then be time-locked to a specific behavioural output^26^,^27^,^42^. Our fibre photometry system used two LEDs at 490 and 405 (Thor Labs), reflected off dichroic mirrors (Semrock, FF495) and coupled into a 400 μm 0.48 NA optical fibre (Thorlabs BFH48–600, important for minimal autofluorescence and signal recovery) using a 40 × 0.48 NA microscope objective (Olympus) and fibre launch (Thorlabs), with the patchcord linked to an implanted 400 μm optical fibre with zirconia sheath. The LED intensity at the interface between the fibre tip and the animal ranged from 30 to 75 μW (but was constant across trials and over days). The power output of the system was tested with a power meter at the start of each experimental session. A real-time signal processor (RX8, Tucker-Davis Technologies, Alachua FL), running software that was custom designed using OpenEx, sinusoidally modulated each laser's output. The two output signals were projected onto a photodetector (Model 2151 Femtowatt Photoreceiver, Newport, Irvine, CA) after which they were separated for analysis. Signals were collected at a sampling frequency of 381 Hz.

### Fibre photometry analysis

Analysis of the signal was accomplished with custom-written MatLab software^42^. The bulk fluorescent signals from each channel were normalized, to compare across animals and experimental sessions. The 405 channel was used as the control channel. GCaMP signals that are recorded at this wavelength are not calcium dependent; thus, changes in signal can be attributed to autofluorescence, bleaching and fibre bending. Accordingly, any fluctuations that occurred in the 405 nm control channels were removed from the 490 nm channel before analysis.

To do this, both signals were first filtered at 40 Hz. A least-squares linear fit was applied to the 405 nm control signal, to align it to the 490 nm signal. The Δ*F*/*F* was then calculated for each behavioral session as: (490 nm signal−fitted 405 nm signal)/(fitted 405 nm signal). We calculated a single baseline fluorescence value, either as the median of the entire trace (which robustly estimated the baseline fluorescence) or by manually defining the baseline during visually identified periods of rest. We calculated the normalized change in fluorescence (Δ*F*/*F*) by subtracting the baseline fluorescence from the fibre fluorescence at each time point and dividing that value by the baseline fluorescence. Peak reward Δ*F*/*F* was calculated as the maximum Δ*F*/*F* value in the paired chamber in a 10 s period around the entry. It is important to note that this window is only applied around trials where the animal is facing the chamber and subsequently enters the chamber.

Behavioural variables, such as paired and unpaired chamber entries, were marked in the signalling traces via the real-time processor as electrical pulses from Noldus Ethovision software. This allowed for precise determinations of the temporal profile of signals in relation to paired and unpaired chamber entries. For each mouse, the time at which the animal entered the drug-paired chamber was determined and a 5 s window around that point was analysed. We subsequently determined the maximum or minimum fluorescent signal that occurred within that window over both consecutive entries and across behavioural tasks/sessions.

Peak analysis to determine frequency and amplitude of the signals was carried out by determining the median average deviation of the corrected/normalized data set and peaks were identified as events that exceeded the median average deviation by 2.91 deviations.

### Western blot hybridization

Bilateral 14-gauge NAc punches were collected on ice after rapid decapitation and then immediately placed on dry ice and stored at −80 °C until use. Protein concentrations of whole-cell lysates were determined using a DC protein assay (Bio-Rad). Within each fraction, equal amounts of protein were mixed with reducing sample buffer containing SDS and dithiothreitol, and samples heated to 95 °C for 5 min. Samples were separated by molecular weight using SDS–PAGE with Criterion 4–15% Tris-HCl, 1.0 mm precast gels (Bio-Rad) and proteins transferred onto Immobilon-P 0.45 μm pore side membranes (Millipore) at 100 V for 1 h at 4 °C in SDS-free transfer buffer containing 15% methanol. Membranes were blocked in Tris-buffered saline containing 5% BSA and 0.1% Tween-20 for 1 h at room temperature and membranes incubated with a polyclonal primary antibody to DAT (Millipore AB2231; 1:1,000), pDAT (Abcam ab28; 1:1,000), TH (Abcam ab112; 1:1,000), pS40 TH (Abcam ab51206; 1:1,000), Erk1/2 (cell signaling 4695S; 1:1,000) or pERK1/2 (Cell Signaling 4370; 1:1,000) in blocking solution overnight at 4 °C. Membranes then were incubated with a peroxidase-conjugated secondary antibody (goat anti-rabbit, Jackson Immunoresearch; 1:50,000) in blocking solution at room temperature for 2 h, washed and developed using chemiluminescent substrate (Thermo Scientific). The amount of protein expressed in each lane was quantified by densitometry using Image J (US National Institutes of Health). Actin levels were used to normalize measurements. For DAT, two bands (at 75 and 55 kDA) are obtained due to different glycosylation states of the mature protein. Each band was quantified separately and the ratio of the 75 kDA band to the 55 kDA band was compared. Images in Fig. 2 and Supplementary Figs 3 and 13 have been cropped for presentation. Full size images are presented in Supplementary Fig. 17.

### Statistics

Graph Pad Prism (version 6, La Jolla, CA, USA) or R were used to statistically analyse data sets and create graphs. Normality of data was assessed and, when data were not normally distributed, non-parametric analyses were performed. Data for CPP across oestrous cycle, VTA dopamine neuron percent spikes within a burst, voltammetrically recorded dopamine release, cocaine effects during fibre photometry recordings, cocaine affinity measurements (*K*_i_), VTA contextual cue responses, baseline VTA activity as measured via fibre photometry, cycle-dependent changes in uptake rates and cycle-dependent changes in *K*_m_ were analysed by a one-way analysis of variance with Tukey's *post-hoc* test. Data of VTA dopamine neuron *in vivo* firing rate, VTA dopamine neuron burst length and NAc contextual cue-responses were analysed using a Kruskal–Wallis *χ*^2^-test because of non-normal distributions followed by Wilcoxon test. Data for phasic stimulations responses across groups, quinpirole concentration response curves, cocaine-induced inhibition of VTA and NAc activity using fibre photometry, cocaine concentration–response curves, cocaine-induced dopamine release and effects of estradiol and CNO during FSCV recordings were analysed by a two-way analysis of variance with Bonferroni *post-hoc* test. Initial male versus female comparisons for CPP and basal VTA electrophysiology were compared using a Student's *t*-test. Correlational analyses were used to compare CPP difference score with cocaine effects in the VTA and NAc, contextual-cue response with difference score and maximal rate of uptake with cocaine affinity (*K*_i_). *P*-levels <0.05 were considered to be statistically significant for all data.

### Data availability

The authors declare that the data supporting the findings of this study are available within the paper and its Supplementary Information files.

## Additional information

**How to cite this article:** Calipari, E. S. *et al*. Dopaminergic dynamics underlying sex-specific cocaine reward. *Nat. Commun.* **8,** 13877 doi: 10.1038/ncomms13877 (2017).

**Publisher's note:** Springer Nature remains neutral with regard to jurisdictional claims in published maps and institutional affiliations.

## Supplementary Material

Supplementary Information Supplementary Figures
