# Supplementary material for: Dopaminergic dynamics underlying sex-specific cocaine reward
Source: Nat Commun. 2017 Jan 10;8:13877. doi: 10.1038/ncomms13877 (PMC5234081; doi:10.1038/ncomms13877)
Supplement: Supplementary Information — Supplementary Figures [file ncomms13877-s1.pdf]

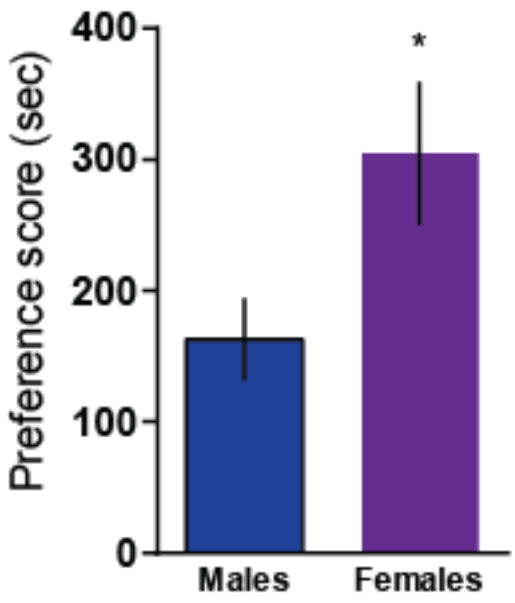

Supplementary Figure 1. Female mice show enhanced conditioned place preference. Both male and female mice develop a CPP for the cocaine-paired chamber (males, one sample t-test, $t_{(5)} = 5.20$, $P < 0.01$; females, one sample t-test, $t_{(9)} = 5.62$, $P < 0.001$). Female mice exhibit a higher preference for the cocaine-paired context as compared to males (Student's t-test; $t_{(13)} = 1.97$, $^*P < 0.05$). Data represented as mean ± S.E.M.

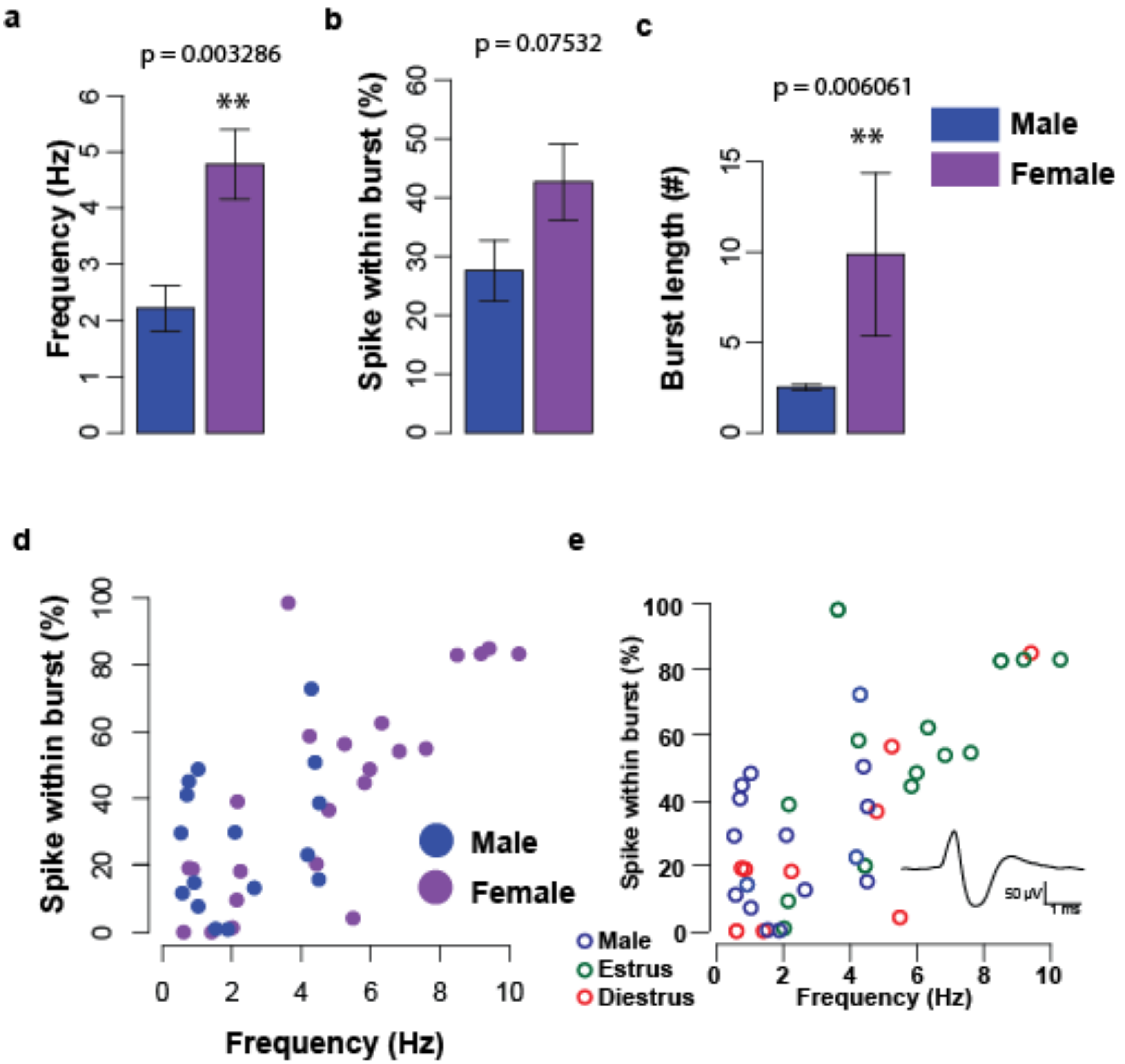

Supplementary Figure 2. Female mice show enhanced basal VTA dopamine activity. Female mice (both estrus and diestrus) exhibit: (**a**) significantly increased firing rates of putative VTA dopamine neurons *(P*<0.05), (**b**) trending increased spikes within VTA dopamine bursts *(P*=0.07532), and (**c**) increased burst length when compared to male mice (*P*<0.05). (**d**) Dot plot showing each recorded neuron's frequency and average % spikes within burst for males and females. (**e**) Dot plot showing each recorded neuron's frequency and average % spikes within burst for males, estrus females and diestrus females. (**e, inset**) Typical triphasic waveform associated with VTA DA neurons during *in vivo* anesthetized single unit recordings. ***P*<0.01, Wilcoxon Signed-Rank Test for non parametric analyses in (**a**) and (**c**). Students t-test for (**b**). Male (*n*=16 neurons); Female (*n*=23 neurons). ** *P*< 0.01. Data represented as mean ± S.E.M.

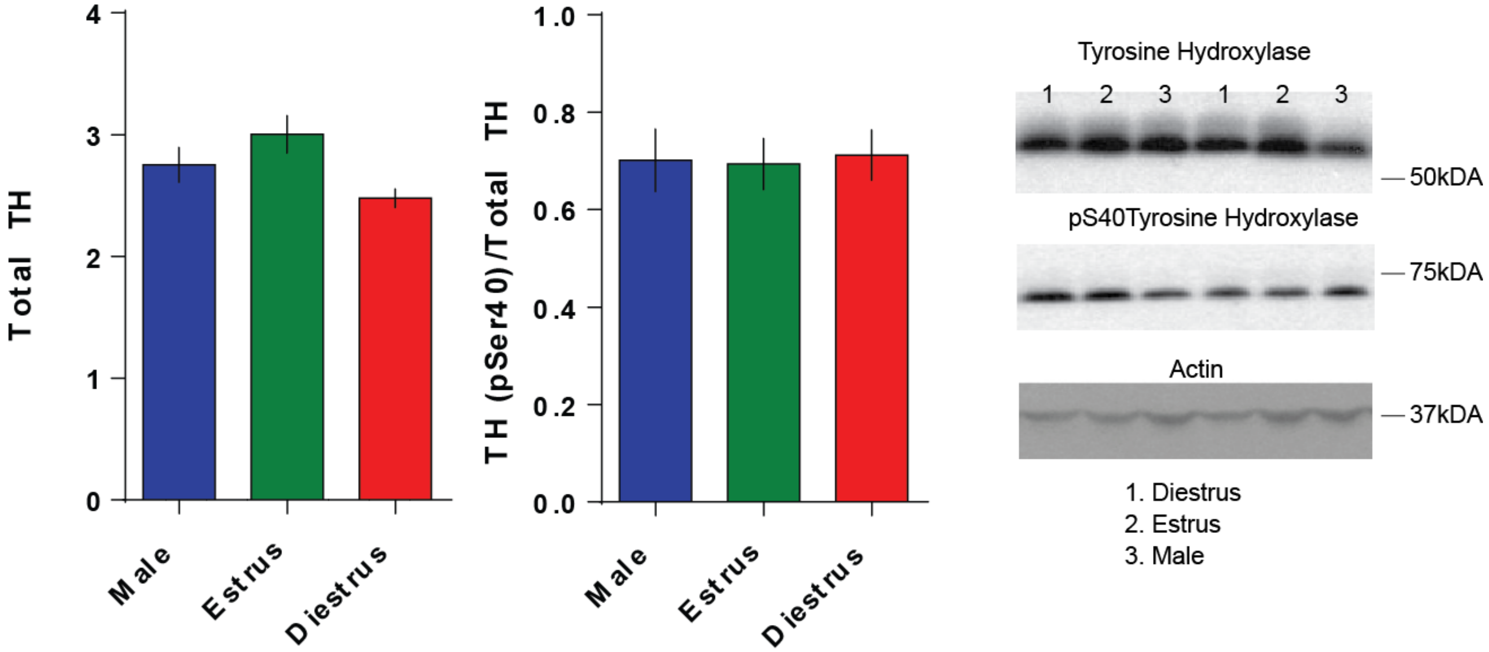

Supplementary Figure 3. Tyrosine hydroxylase levels and activity are unchanged. There were no significant changes in total tyrosine hydroxylase levels (left; One Way ANOVA $F_{(2, 21)} = 0.51$; $P = 0.607$) or in the amount of tyrosine hydroxylase phosphorylated at the S40 site (One Way ANOVA $F_{(2, 21)} = 0.02678$; $P = 0.973$). Data represented as mean ± S.E.M.

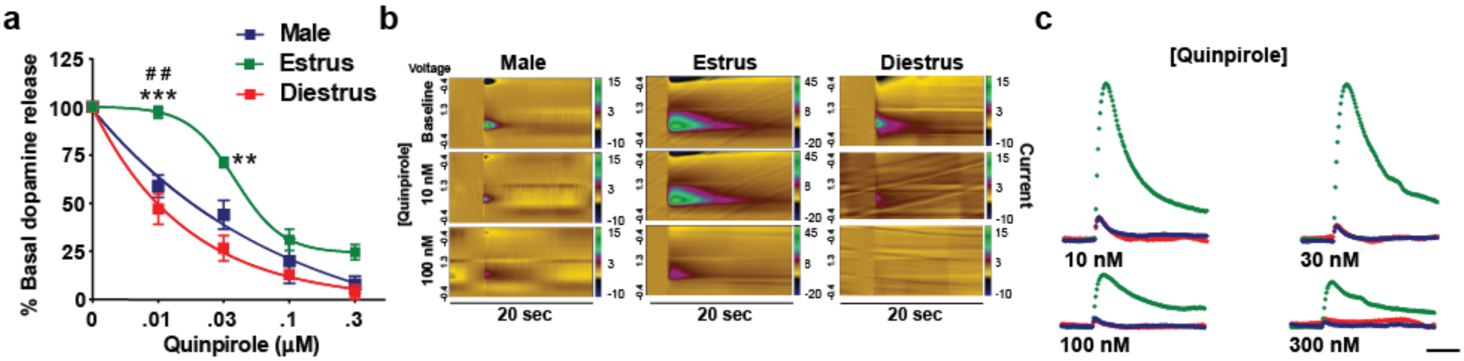

Supplementary Figure 4. D2-autoreceptor feedback mechanisms are blunted during estrus. (**a**) Autoreceptor function was assessed by determining the ability of the D2 agonist, quinpirole, to reduce dopamine release over a concentration-response curve. The IC50 for quinpirole was reduced in estrus females (left) (two-way ANOVA; $F_{(8, 16)} = 4.096$, $P<0.01$; ** $P< 0.01$, *** $P< 0.001$, ## $P<0.01$). (b) Color plots of dopamine release. (**c**) Current versus time plots in each group. ** $P< 0.01$, *** $P< 0.001$ vs diestrus; ## $P< 0.01$ vs males. Data represented as mean ± S.E.M.

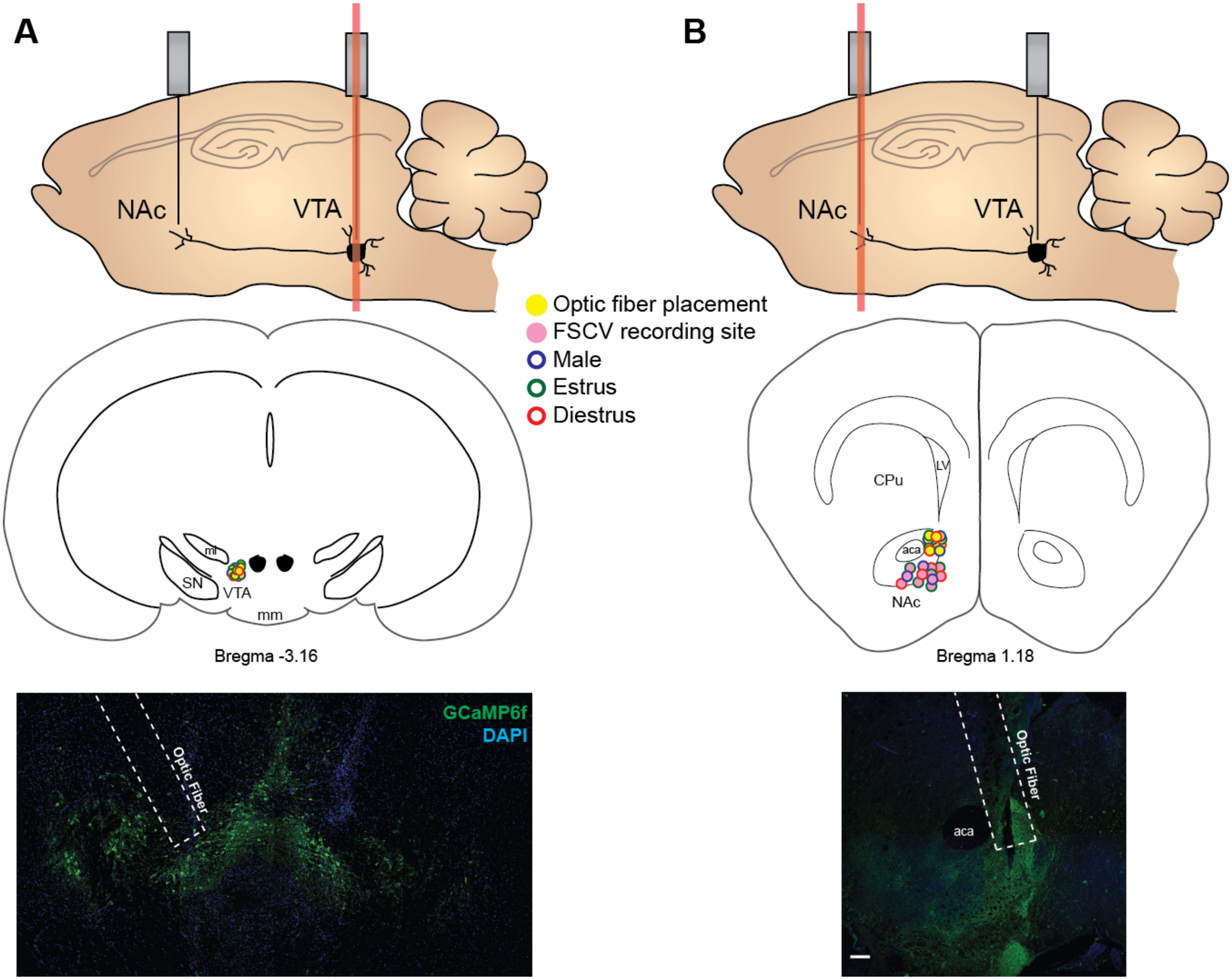

Supplementary Figure 5. Morphological and immunohistochemical validation. (**a**) Dots in VTA represent unilateral optic fiber placement for each mouse used in VTA fiber photometry recordings (top and middle) (yellow center). Representative immunohistochemical validation of AAV-CamKII-GCaMP6f-GFP expression in the VTA with optic fiber track (bottom). (**b**) Dots in NAc represent unilateral optic fiber placement (yellow center) for each mouse used in VTA terminal recordings in NAc core fiber photometry recordings (top and middle). Also shown are dots where fast-scan cyclic voltammetry (FSCV) recordings (pink center) were performed (middle). Representative immunohistochemical validation of AAV-CamKII-GCaMP6f-GFP expression in VTA terminals in the NAc with optic fiber track (bottom).

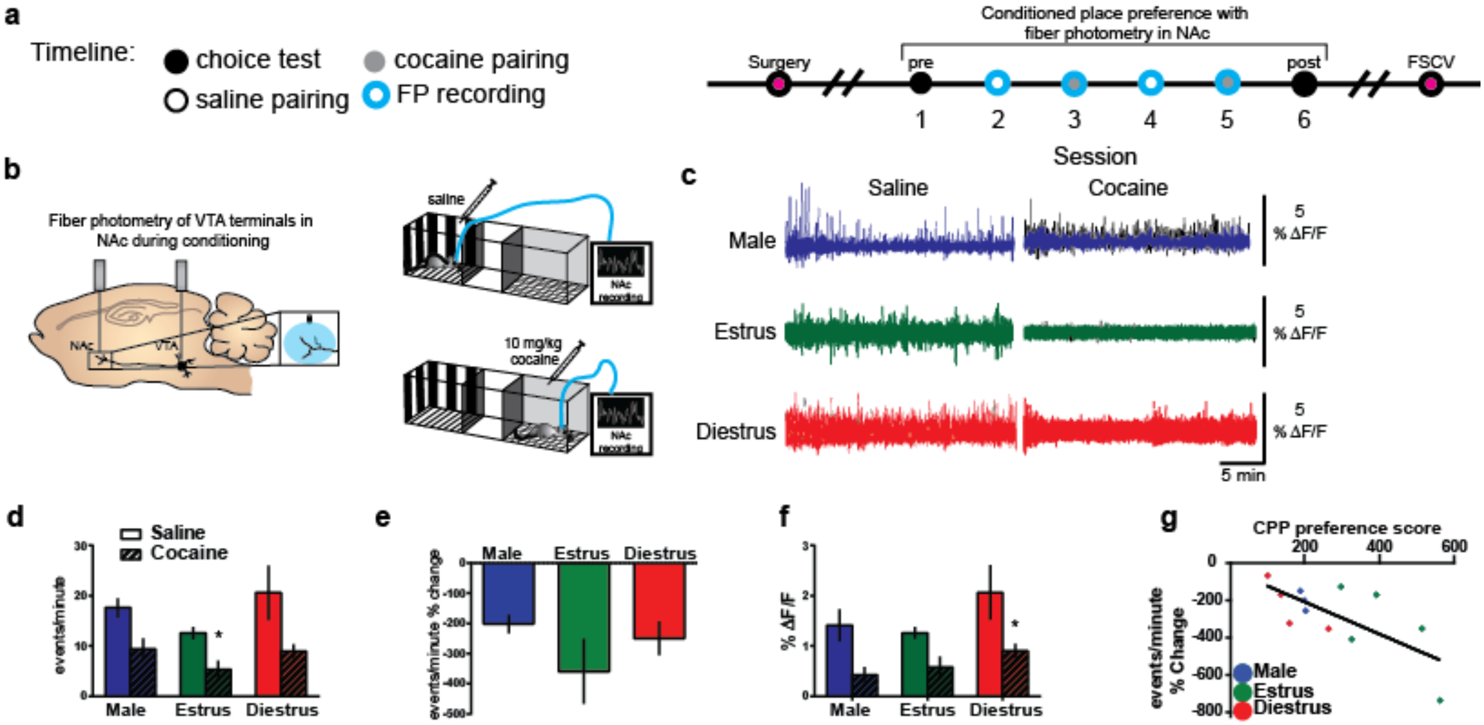

Supplementary Figure 6. Fiber photometry recordings of VTA terminals in the NAc during cocaine conditioning. (a) Timeline of experiments. (**b**) Schematic of fiber photometry recordings from VTA terminals located in the NAc. (**c**) Representative Ca2+ imaging traces from male, estrus females and diestrus female mice during saline and cocaine conditioning. (**d**) Cocaine reduces the frequency of VTA terminal activity in the NAc (two way ANOVA; $F_{(1,10)}$= 16.34, P<0.05; *P<0.05 vs saline). (**e**) Cocaine induced reductions in activity across groups. (**f**) Cocaine-induced changes in the amplitude of Ca2+ events (two way ANOVA; $F_{(1,10)}$=14.31, P<0.01; *P<0.05 vs saline). (**g**) Correlation between change in Ca2+ transient activity of VTA terminals in the NAc and CPP (r=0.4984; P<0.05). Data represented as ± SEM

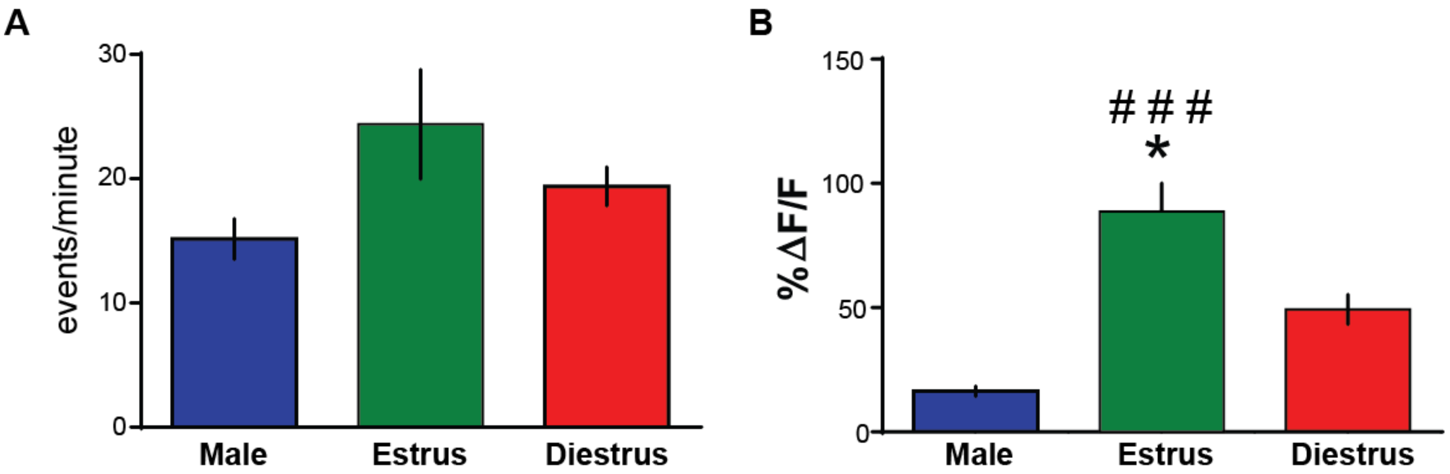

Supplementary Figure 7. Baseline activity measurements from fiber photometry recordings conducted for VTA cell bodies, (**a**) Frequency of activity in male and estrus and diestrus female mice measured during the first saline conditioning. (**b**) Baseline activity amplitude changes in the same three groups of mice measured during the first saline conditioning ($F_{(2, 10)} = 15.12$, $P < 0.001$). *P< 0.01 estrus vs diestrus, ###P<0.001 estrus vs male. Data represented as mean ± S.E.M.

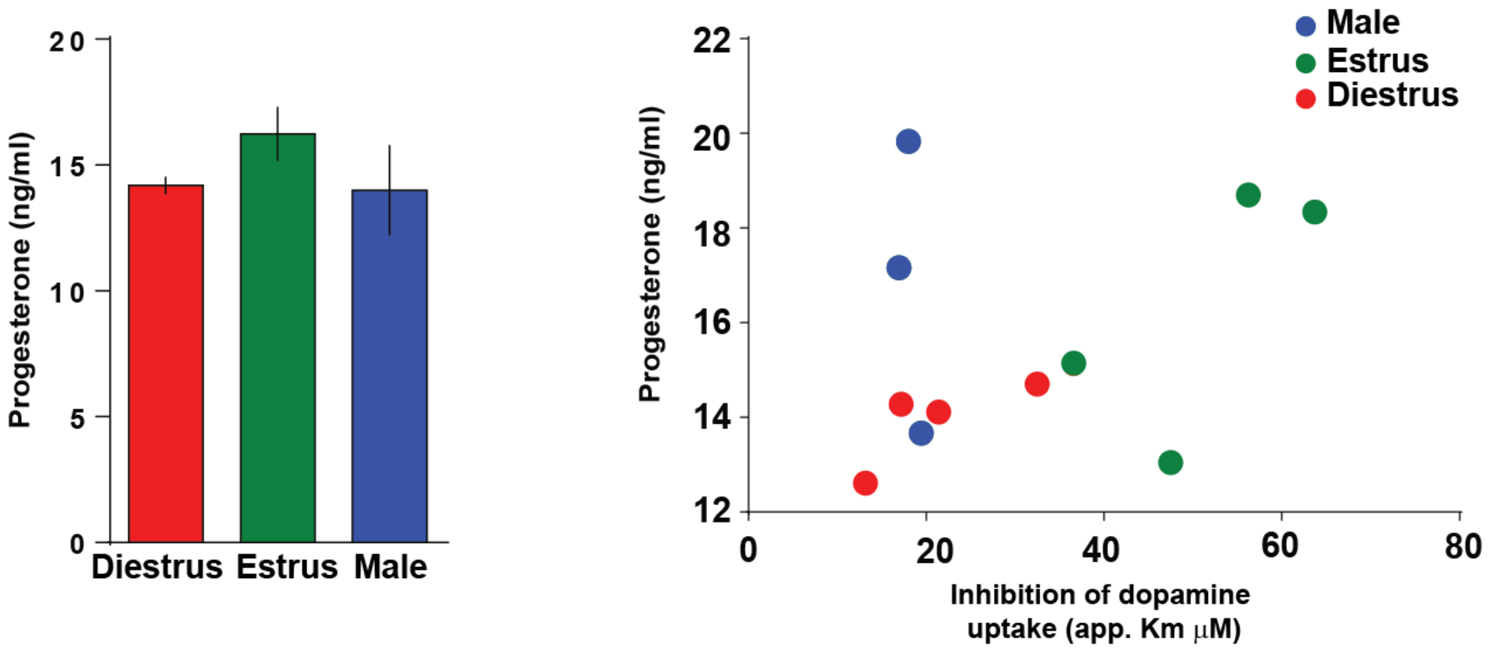

Supplementary Figure 8. Circulating serum progesterone levels are not predictive of changes in cocaine potency. (**a**) Serum progesterone levels from males, estrus female and diestrus females used in FSCV recordings ($F_{(2, 11)}$ = 1.788, P = 0.21). (**b**) Serum progesterone levels versus cocaine potency at the dopamine transporter as measured by apparent Km (right) (r = 0.35, p = 0.29).

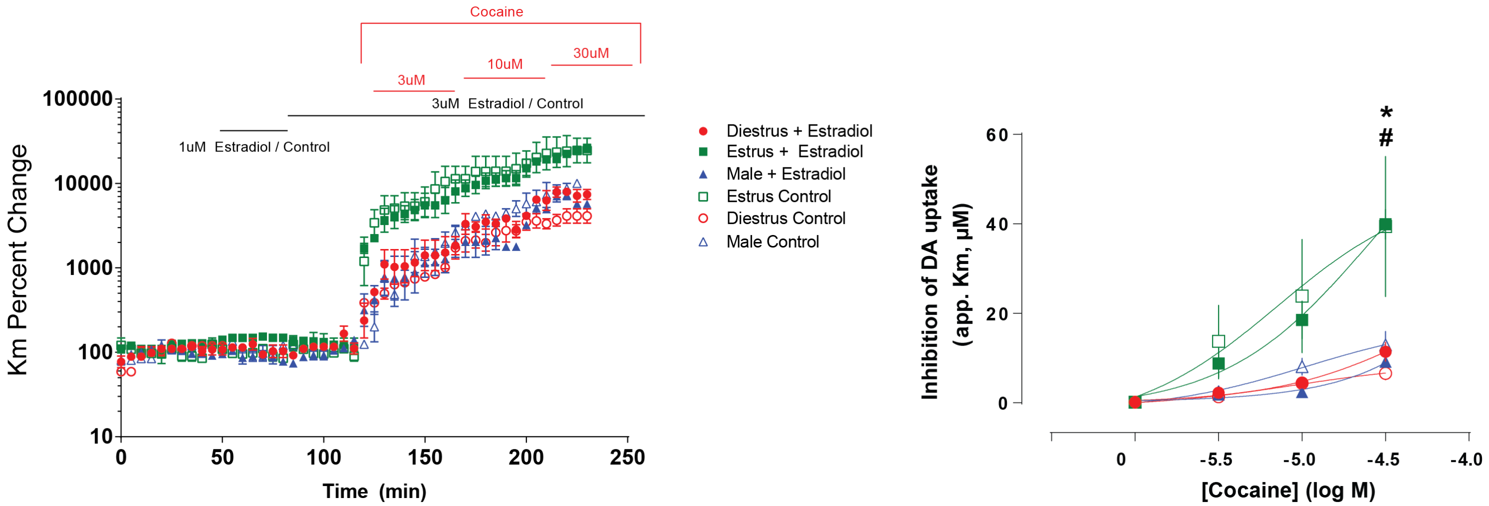

Supplementary Figure 9. No effect of estradiol on cocaine potency directly at dopamine terminals. Voltammetric dopamine recordings were done in the nucleus accumbens of estrus females, diestrus females, and males. Estradiol had no effect on dopamine kinetics. Cocaine potency was enhanced during estrus ($F_{(5, 23)} = 8.76$, $P < 0.0001$; **P*<0.05 estrus vs diestrus, #*P*<0.05 estrus vs male); however, there was no effect in any group of estradiol application to the dopamine terminals. Data represented as + SEM.

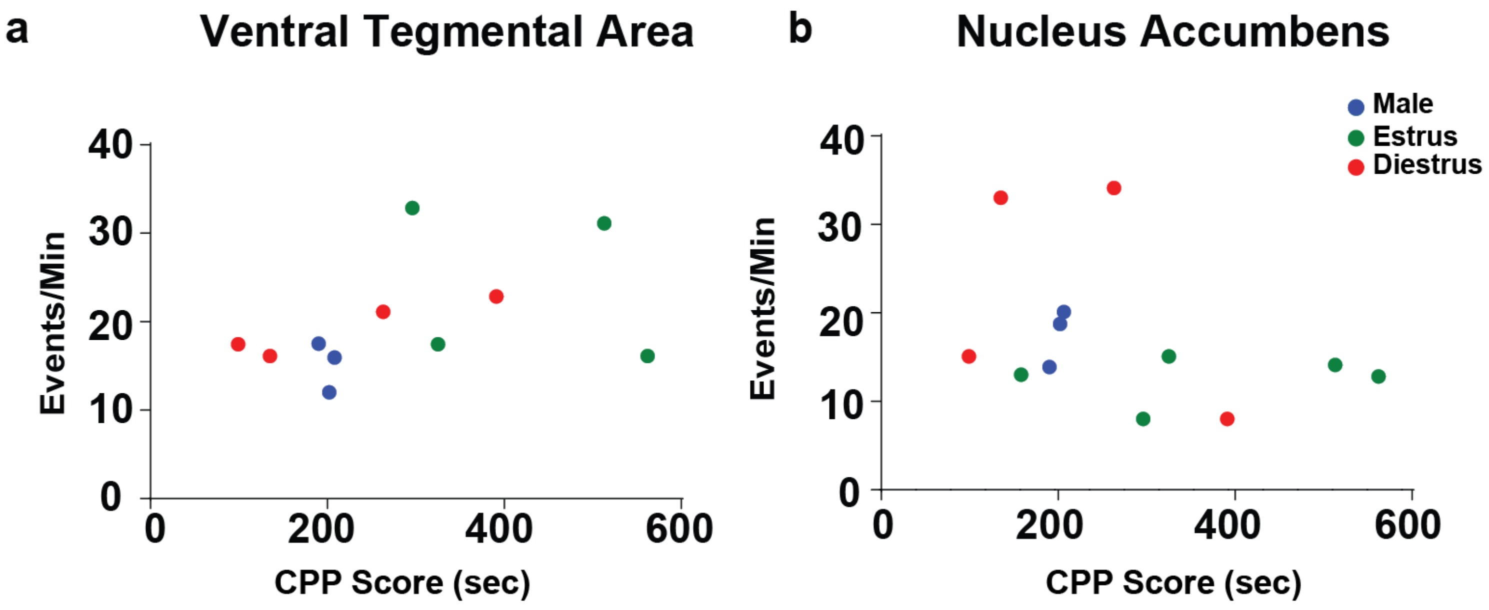

Supplementary Figure 10. No correlation between VTA event rate and conditioned place preference. The event rate of the VTA and NAc at baseline were compared to the formation of conditioned place preference in each animal. **(a)** Correlation analysis of events per minute in the VTA with conditioned place preference (r = 0.42, P = 0.2). (**b**) Correlation analysis of events per minute in the terminals in the NAc with conditioned place preference (r = -0.35, P = 0.28).

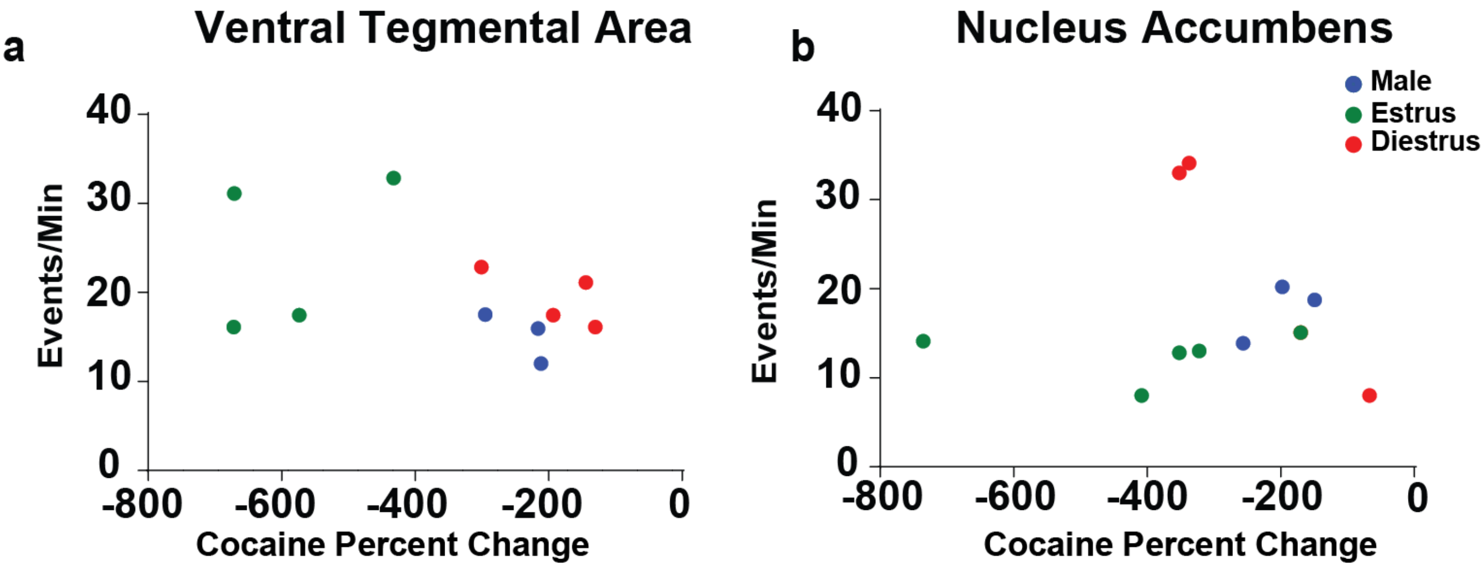

Supplementary Figure 11. No correlation between VTA baseline event rate and cocaine's molecular effects. The event rate of the VTA and NAc at baseline were compared to the ability of cocaine to suppress activity within each region. **(a)** Correlation analysis of events per minute in the VTA with the pharmacological effect of cocaine as expressed by the percent change in events/min induced by cocaine injection preference (r = - 0.41, P = 0.21). (**b**) Correlation analysis of events per minute in the terminals in the NAc with the pharmacological effect of cocaine (r = - 0.07, P = 0.83).

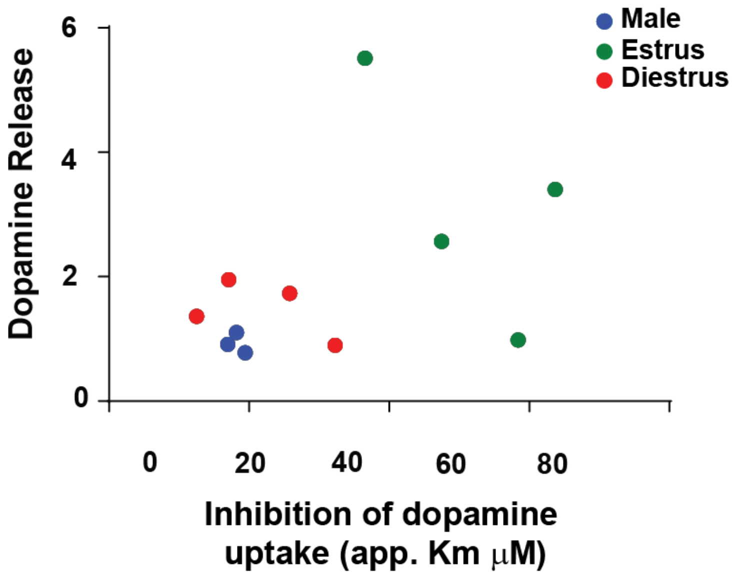

Supplementary Figure 12. No correlation between dopamine release and cocaine potency. Correlation analysis comparing basal dopamine release in µM with the pharmacological effect of cocaine as measured by apparent Km. No correlation was observed (r = 0.39, *P* = 0.23).

.

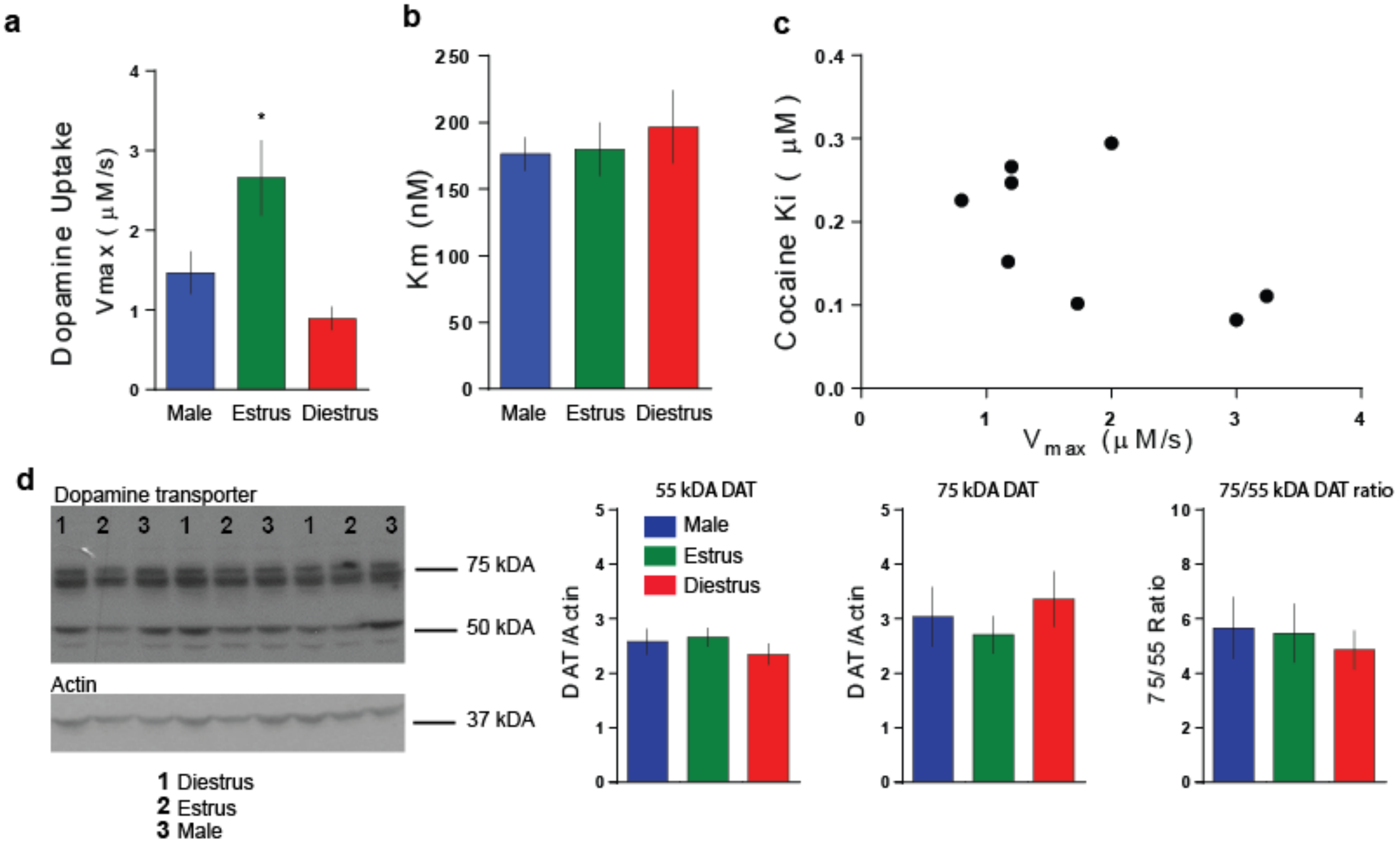

Supplementary Figure 13. Changes in cocaine potency at DAT are not related to basal differences in transporter function or levels. (a) Maximal rate of dopamine uptake across groups showing an increase during estrus (one way ANOVA $F_{(2, 6)}$ = 7.78, *P*<0.05, **P*<0.05). **(b)** Basal affinity of dopamine for DAT as a measure of K_m. There were no differences between groups (one way ANOVA $F_{(2, 6)}$ = 0.27, *P*=0.77). **(c)** Lack of correlation (r= -0.59) between maximal rate of dopamine uptake and the affinity of cocaine for DAT, confirming that changes in cocaine potency at DAT were separate from differences in basal DAT function. **(d)** Western blotting showing no difference in total levels of DAT protein among male and estrus and diestrus female mice at baseline or after cocaine CPP (55kDA DAT: one way ANOVA $F_{(2, 21)}$ = 0.48, *P* = 0.62; 75kDA DAT: one way ANOVA $F_{(2, 21)}$ = 0.68, *P* = 0.52; 55/75kDA DAT: one way ANOVA $F_{(2, 21)}$ = 0.18, *P* = 0.84). Data represented as mean ± S.E.M.

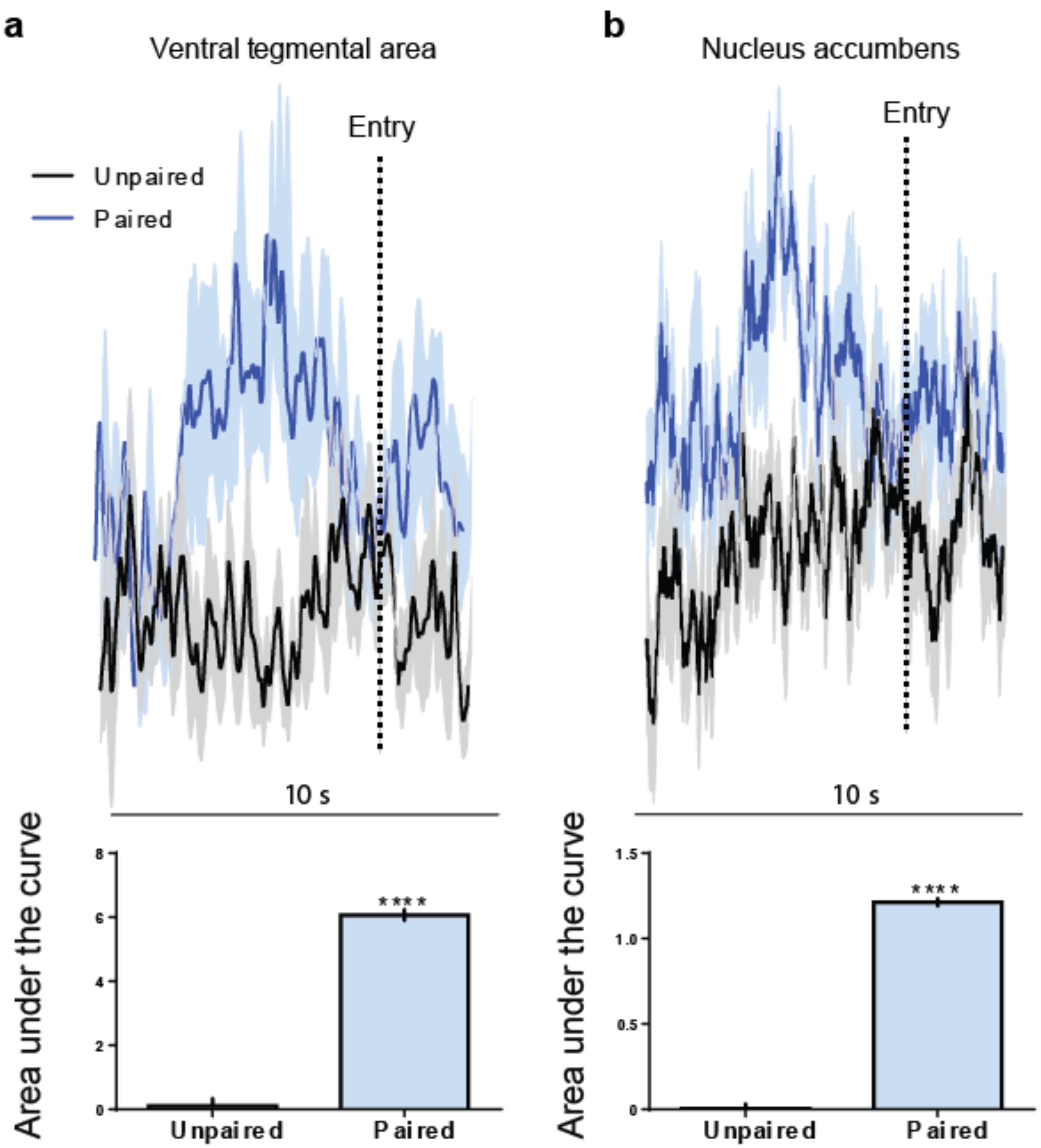

Supplementary Figure 14. VTA and NAc Ca^{2+} responses to cocaine-associated, but not neutral, contextual cues. Fiber photometry Ca^{2+} imaging in the VTA (Student's *t* test $t_{(17)}$=20.74, ****P< 0.00001) **(A)** and VTA terminals in the NAc (Student's *t* test $t_{(17)}$=32.30, ****P< 0.00001) **(B)** during exposure to contextual cues associated with cocaine or saline (neutral cue). Data represented as mean ± S.E.M.

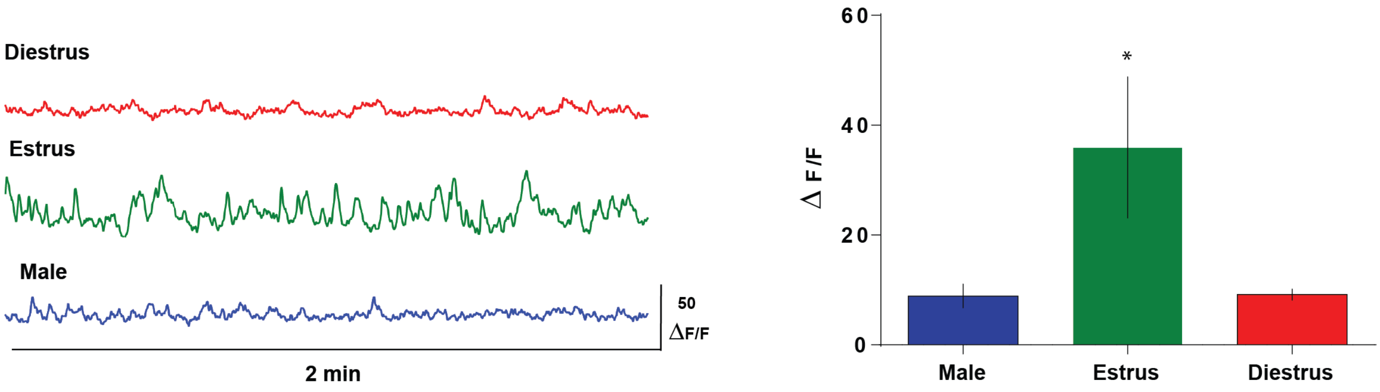

Supplementary Figure 15. Enhanced VTA dopamine neuron activity during estrus. (a) Sample traces of VTA dopamine neuron calcium activity in male mice and female mice during estrus and diestrus. **(b)** Average amplitude of VTA dopamine neuron calcium events across groups (one-way ANOVA $F_{(2,\ 8)}$ = 5.798, *P* = 0.02, **P*=0.05). Data represented as mean ± S.E.M.

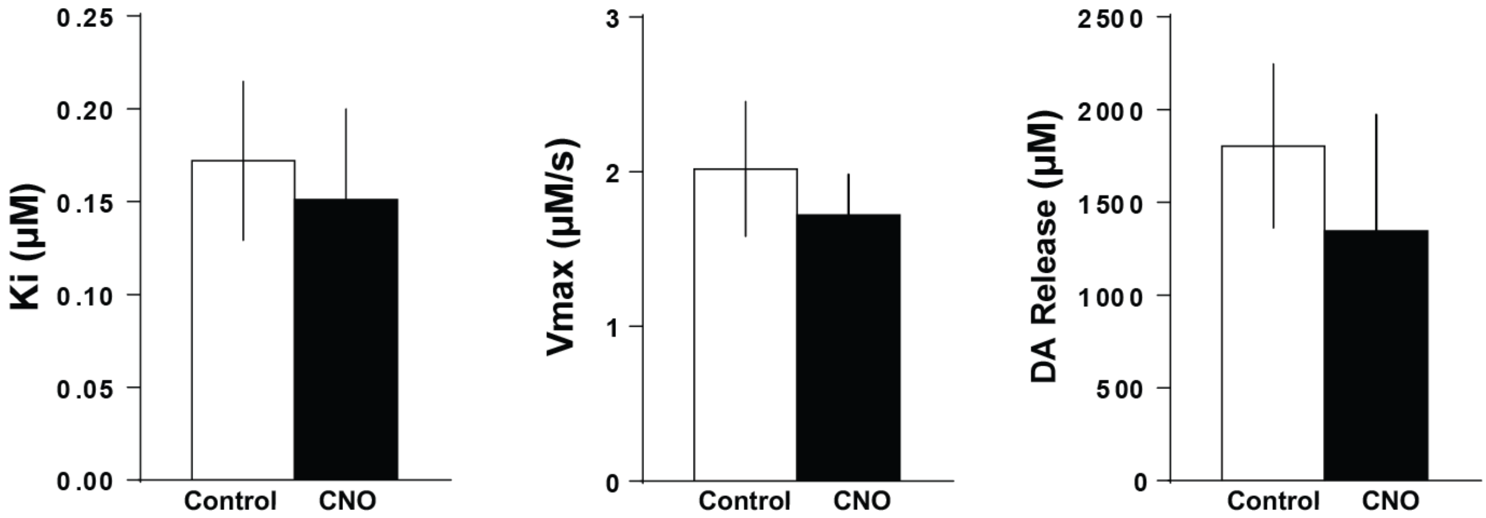

Supplementary Figure 16. CNO in the absence of DREADDs does not alter dopamine system function. (left) Ki values (Student's *t* test, $t_{(6)}$=0.2575 *P* = 0.42) (middle) Uptake rate values (Student's *t* test, $t_{(6)}$=0.340 *P* = 0.35). (right) Dopamine release (Student's *t* test, $t_{(9)}$=0.56 *P*=0.3). Data represented as mean ± S.E.M.

-

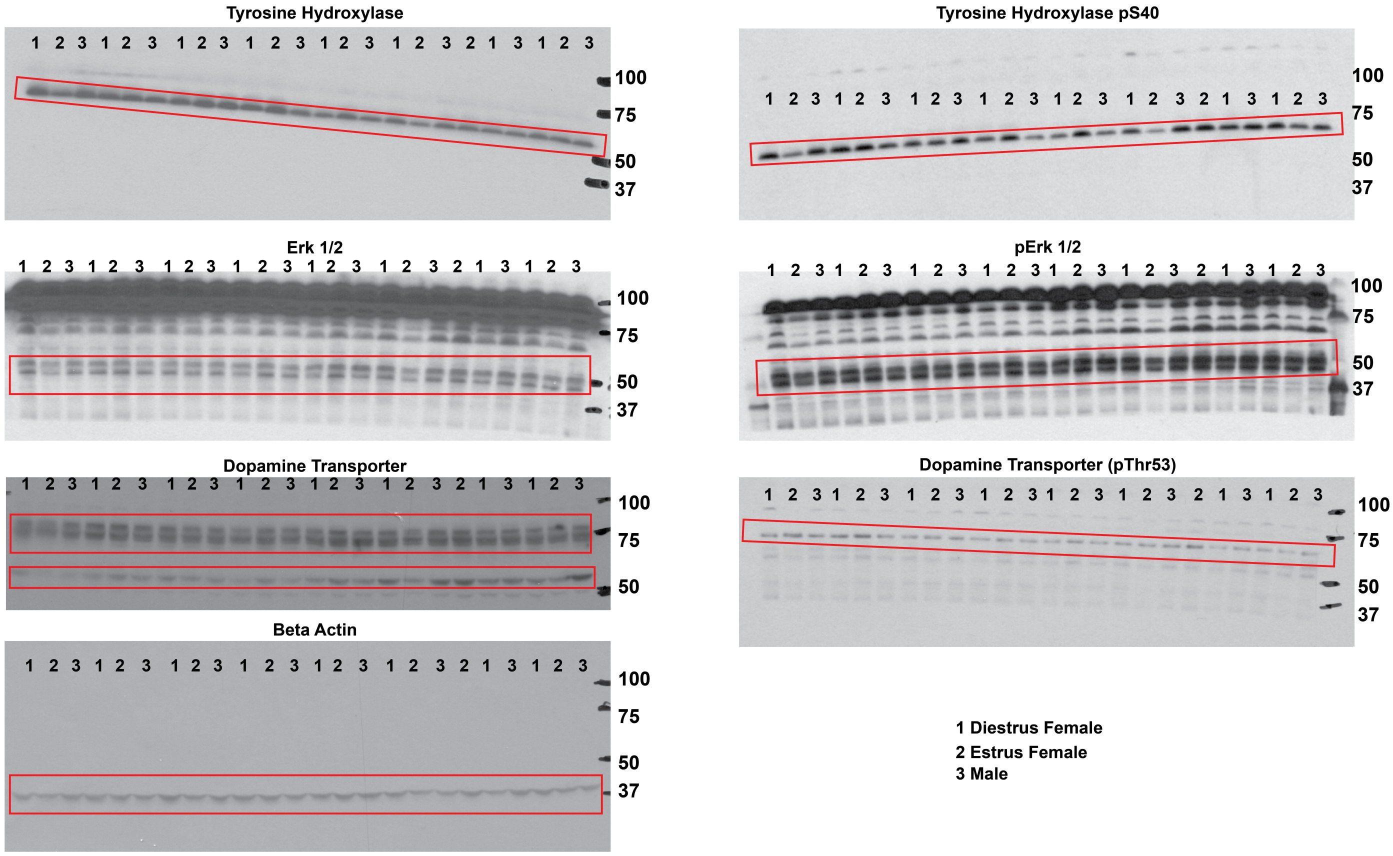

Supplementary Figure 17. Western Blots. Full western blot images from each protein assayed. Red boxes denote the band analyzed.
